# Nanocatalytic Tumor Therapy by Biomimetic Dual Inorganic Nanozyme‐Catalyzed Cascade Reaction

**DOI:** 10.1002/advs.201801733

**Published:** 2018-11-12

**Authors:** Shanshan Gao, Han Lin, Haixian Zhang, Heliang Yao, Yu Chen, Jianlin Shi

**Affiliations:** ^1^ State Key Laboratory of High Performance Ceramics and Superfine Microstructure Shanghai Institute of Ceramics Chinese Academy of Sciences Shanghai 200050 P. R. China; ^2^ University of Chinese Academy of Sciences Beijing 100049 P. R. China; ^3^ Department of Ultrasound Fudan University Shanghai Cancer Center Department of Oncology Shanghai Medical College Fudan University Shanghai 200032 P. R. China

**Keywords:** cascade reaction, nanocatalytic biomedicine, nanocatalytic tumor therapy, nanomedicine, nanozymes

## Abstract

Emerging nanocatalytic tumor therapies based on nontoxic but catalytically active inorganic nanoparticles (NPs) for intratumoral production of high‐toxic reactive oxygen species have inspired great research interest in the scientific community. Nanozymes exhibiting natural enzyme‐mimicking catalytic activities have been extensively explored in biomedicine, mostly in biomolecular detection, yet much fewer researches are available on specific nanocatalytic tumor therapy. This study reports on the construction of an efficient biomimetic dual inorganic nanozyme‐based nanoplatform, which triggers cascade catalytic reactions for tumor microenvironment responsive nanocatalytic tumor therapy based on ultrasmall Au and Fe_3_O_4_ NPs coloaded dendritic mesoporous silica NPs. Au NPs as the unique glucose oxidase‐mimic nanozyme specifically catalyze β‐D‐glucose oxidation into gluconic acid and H_2_O_2_, while the as produced H_2_O_2_ is subsequently catalyzed by the peroxidase‐mimic Fe_3_O_4_ NPs to liberate high‐toxic hydroxyl radicals for inducing tumor‐cell death by the typical Fenton‐based catalytic reaction. Extensive in vitro and in vivo evaluations have demonstrated high nanocatalytic‐therapeutic efficacy with a desirable tumor‐suppression rate (69.08%) based on these biocompatible composite nanocatalysts. Therefore, this work paves a way for nanocatalytic tumor therapy by rationally designing inorganic nanozymes with multienzymatic activities for achieving high therapeutic efficacy and excellent biosafety simultaneously.

Tumor microenvironment (TME) is generally known to feature abundant unique characteristics such as acidity,^1^ hypoxia,^2^ inflammation,^3^ and overproduced hydrogen peroxide.^4^ Based on the unique tumor physiological microenvironment and the rising nanotechnology, a number of promising nanotheranostic strategies of designing TME‐specific/responsive nanoplatforms for efficient tumor therapy and precise bioimaging diagnosis have been extensively explored and developed.^5^ Tumor cells produce large amounts of hydrogen peroxide (H_2_O_2_) by the overexpressed superoxide dismutase (SOD) through a catalytic process by superoxide ions as generated from mitochondria.^6^ As a typical feature of TME, the overexpressed H_2_O_2_ has thus been exploited for triggering responsive drug‐releasing in chemotherapy or producing endogenous O_2_ for oxygen‐favored cancer therapy such as photodynamic therapy,^7^ sonodynamic therapy,^8^ or radiotherapy.^9^

As an emerging efficient cancer‐therapeutic modality, the proof‐of‐concept nanocatalytic tumor therapy as first proposed by our group^10^ utilizes intratumoral in situ catalytic chemical reactions to produce oxidative stress for inducing cancer‐cell death by damaging intracellular biomolecule substances such as proteins, lipids, and DNA.^11^ A typical strategy of the proposed nanocatalytic tumor therapy is the representative chemodynamic therapy,^12^ which converts less reactive endogenous H_2_O_2_ into the most harmful reactive oxygen species (ROS), hydroxyl radicals (·OH) under mildly acidic TME via an intratumoral Fenton or Fenton‐like reaction by metal ions.^13^ Such an endogenous but direct chemical energy conversion strategy uses no external energy input such as laser, ultrasound, or magnetic field, thus avoiding the limitations of low tissue‐penetration depth and nonspecificity of these external triggers on inducing cancer‐cell death.

Based on the fact that the intratumoral H_2_O_2_ concentration, generally believed to be 50–100 × 10^−6^ m,[[qv: 4b]] is too low to produce desirable and sufficient amount of hydroxyl radicals for inducing satisfactory nanocatalytic‐therapeutic efficacy via Fenton or Fenton‐like reactions, natural glucose oxidase (GOx) was introduced to elevate the intratumoral H_2_O_2_ concentration through natural catalytic oxidation of intratumoral glucose to H_2_O_2_ and gluconic acid.^14^ However, natural GOx possesses several intrinsic drawbacks such as high cost in preparation and purification, and relatively low operational stability, which will undoubtedly hamper its practical biomedical application under complicated and harsh physiological environments.^15^ Therefore, exploring and developing natural GOx alternatives with much‐enhanced stability and lowered cost is highly desirable and necessary.

Recently, the merging of biology with nanotechnology has motivated extensive research fever for designing functional nanoplatforms that exhibit unique natural enzyme‐mimic catalytic activities for a broad range of biomedical applications.^16^ As the promising alternatives for natural enzymes, catalytically active nanomaterials, known as the so‐called “artificial enzymes” or “nanozymes,” have demonstrated numbers of merits over natural enzymes, such as facile fabrication, low cost, and robust stability against severe conditions.^17^ So far, a variety of nanomaterials, such as carbon‐based nanoparticles (NPs),^18^ metal NPs,^19^ and metal oxides NPs[[qv: 16a,20]] have been discovered to possess unique enzyme‐mimic catalytic activities, which are extensively used in numerous fields, including biomolecular detection,^21^ biosensor,^22^ antibacterial applications,^23^ immunoassays,^24^ cancer diagnostics and therapy,^25^ and environmental monitoring.^26^ Till now, an exceedingly large number of reports on peroxidase (POD)‐mimic nanozymes have been reported, while other kinds of enzyme‐mimic NPs, such as GOx‐mimic nanozymes, glutathione peroxidase‐mimic nanozymes, or SOD‐mimic nanozymes, have been rarely explored. Fortunately, Rossi and co‐workers discovered that Au NPs could catalyze the oxidation of glucose to H_2_O_2_ and glucono delta‐lactone (GDL) under the presence of dissolved oxygen, which was very similar to the reaction as catalyzed by GOx, suggesting that Au NPs could serve as a mimic for GOx.^27^ Since then, owing to the unique GOx‐mimic activity, Au NPs have been explored for diverse applications, most of which involve biomolecule (DNA, glucose, etc.) detection, while leaving many hidden applications to be discovered.

Bearing the unique GOx‐mimicking catalytic activity of Au NPs and POD‐mimicking catalytic performance of Fe_3_O_4_ NPs, we herein report, for the first time, on a biomimetic dual inorganic nanozyme‐triggered TME‐responsive cascade catalytic reaction for efficient nanocatalytic tumor therapy based on an “all‐inorganic biocompatible nanosystem”, without employing any toxic chemical drug (**Scheme** 1). Ultrasmall and highly dispersed Au NPs and Fe_3_O_4_ NPs were successively integrated into the large pore channels of dendritic mesoporous silica NPs (DMSN NPs) to construct the composite nanoplatform, i.e., DMSN‐Au‐Fe_3_O_4_ NPs. After further modification with polyethylene glycol (PEG) molecules for improved biocompatibility and physiological stability, DMSN‐Au‐Fe_3_O_4_ NPs could accumulate into tumor tissue via the typical enhanced permeability and retention (EPR) effect, triggering the intratumoral TME‐responsive cascade catalytic reaction.

**Scheme 1:**
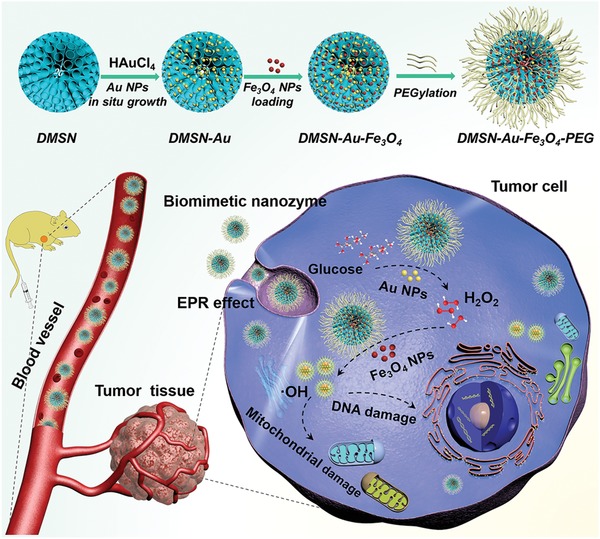
Schematic illustration of “toxic‐drug‐free” nanocatalytic tumor therapy by biomimetic inorganic nanomedicine‐triggered cascade catalytic reaction. The composite nanoplatform with dual inorganic nanozyme activity was fabricated by the sequential loading of Au NPs as GOx‐mimic nanozyme and Fe_3_O_4_ NPs as POD‐mimic nanozyme into the large mesopores of DMSN NPs followed by PEGylation. The therapeutic process by cascade catalytic chemical reaction includes the initial GOx‐mimicking Au nanozyme‐mediated catalytic oxidation reaction of glucose into H_2_O_2_, which is further utilized as the reactant for the POD‐mimicking Fe_3_O_4_ nanozyme‐based catalytic Fenton reaction to produce highly toxic ·OH and subsequently induce tumor‐cell apoptosis.

Acquiring an unusual anaerobic glycolytic behavior to support their metabolism and proliferation, tumor cells demand nutrients, essentially glucose, in a hysterical manner.^28^ Therefore, in TME, masses of glucose molecules are transported and accumulated, which provides the possibility of initiating catalytic reaction by utilizing glucose molecules. The intratumoral cascade catalytic reaction is initially triggered by Au NPs, the GOx mimic inorganic nanozyme which catalyzes the intratumoral glucose oxidation, generating large amounts of H_2_O_2_ molecules to serve as the substrate for the subsequent catalytic reaction where H_2_O_2_ is disproportionated by the coloaded ultrasmall Fe_3_O_4_ NPs via the typical Fenton catalytic reaction, liberating high‐toxic hydroxyl radicals to effectively induce tumor‐cell death. DMSN‐Au‐Fe_3_O_4_ NPs as the potent nanoplatforms are capable of realizing the endogenous cascade reaction for TME‐specific and effective nanocatalytic tumor therapy, in a noninvasive and “toxic‐drug‐free” way, benefiting from the robust biomimetic nanozymes. It is noted that such a cascade reaction with TME acidity‐responsiveness would not be triggered under the neutral condition in normal tissue microenvironment, guaranteeing the high tumor‐specificity and therapeutic biosafety.

DMSN NPs with unique central‐radial pore structures, serving as the robust nanosupports for small NPs, were synthesized by an anion‐assisted approach based on the Stöber mechanism and sol–gel chemistry.^29^ A high density of highly dispersed and ultrasmall particle‐sized Au NPs was confined into the large mesopores of DMSN NPs via an in situ reduction reaction of HAuCl_4_ to produce DMSN‐Au NPs, followed by the collection and integration of ultrasmall Fe_3_O_4_ NPs into the large mesopores of DMSN NPs to fabricate the final composite nanoplatform DMSN‐Au‐Fe_3_O_4_ NPs (**Figure** 1a). Transmission electron microscopic (TEM) images show that DMSN NPs exhibit the unique topology of central‐radial dendritic structure with a uniform diameter of about 140 nm (Figure 1b and inset; Figure S1, Supporting Information). Scanning electron microscopic (SEM) images exhibit the obvious wrinkle structure of DMSN NPs, and their corresponding chemical composition was further confirmed by element mapping of Si and O elements (Figure S2, Supporting Information). The Brunauer–Emmett–Teller surface area and the total pore volume of DMSN NPs were measured to be 197.5 m^2^ g^−1^ and 0.9 cm^3^ g^−1^, respectively, with an average pore diameter of 23.3 nm according to the nitrogen adsorption–desorption isotherm and corresponding pore‐size distribution (Figure S3, Supporting Information). Such a unique branched structure with large pore size and highly accessible surface area of DMSN NPs is highly in favor of the subsequent growth of ultrasmall Au NPs and deposition of Fe_3_O_4_ NPs. After functionalized with amine groups by aminopropyltriethoxysilane (APTES) to provide anchoring sites for Au NPs, the mesopores' surface of DMSN NPs was grown with Au NPs via an in situ reduction of auric ions by NaBH_4._ TEM and SEM images reveal that ultrasmall Au NPs of ≈1.5 nm in diameter have been well dispersed and immobilized within the mesopores channels of DMSN NPs (Figure 1c,d; Figure S4, Supporting Information), and the elemental mappings of Si, O, and Au elements for DMSN‐Au NPs demonstrate the uniform distribution of Au NPs within DMSN's matrix.

**Figure 1:**
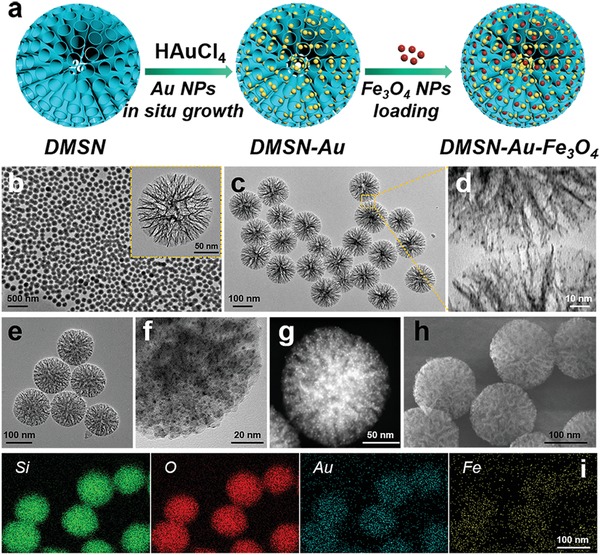
Structural and compositional characterizations of DMSN NPs, DMSN‐Au NPs, and DMSN‐Au‐Fe_3_O_4_ NPs. a) Schematic diagram for the fabrication of DMSN‐Au‐Fe_3_O_4_ NPs. TEM image of b) DMSN NPs, c,d) DMSN‐Au NPs at varied magnifications, e,f) DMSN‐Au‐Fe_3_O_4_ NPs at different magnifications. g) HADDF image, h) SEM image, and i) corresponding element mappings (for Si, O, Au, and Fe) of DMSN‐Au‐Fe_3_O_4_ NPs.

Subsequently, uniform and 1.5 nm sized Fe_3_O_4_ NPs were initially synthesized by a pyrolysis methodology (Figure S5, Supporting Information) and then firmly confined into DMSN‐Au NPs by soaking them in DMSN‐Au NPs‐dispersed dimethyl sulfoxide (DMSO) solution for 24 h to obtain the DMSN‐Au‐Fe_3_O_4_ NPs. After the integration with Au and Fe_3_O_4_ NPs, the unique morphology and dendritic structure of DMSN NPs were well preserved without significant deformation (Figure 1e). High resolution TEM image (Figure 1f) clearly demonstrates that the small Au and Fe_3_O_4_ NPs, both less than 2 nm in diameters, have been uniformly decorated within the mesopore structure of DMSN NPs. The relatively bright area with strong signal intensity in the high angle annular dark‐field scanning TEM image (Figure 1g) indicates the presence and homogeneous distributions of high‐Z Au and Fe_3_O_4_ NPs. SEM image (Figure 1h) and corresponding elemental mappings (Figure 1i) of Si, O, Au, Fe elements also demonstrate the homogeneous distributions of Au and Fe_3_O_4_ NPs into DMSN NPs. Similarly, DMSN‐Fe_3_O_4_ NPs were synthesized for the following experiments at in vitro solution and intracellular levels. SEM and TEM images, and the corresponding elemental mappings of Si, O, Fe elements also confirm the uniform distribution of Fe_3_O_4_ NPs into the DMSN's mesopores in DMSN‐Fe_3_O_4_ NPs (Figure S6, Supporting Information).

X‐ray diffraction (XRD) patterns further verify the formation of Fe_3_O_4_ NPs and metallic Au NPs anchored on DMSN NPs (Figure S7, Supporting Information). The diffraction peaks of Fe_3_O_4_ NPs at 2θ of 18.9°, 29.7°, 35.4°, and 63.1° were respectively indexed to the (111), (220), (311), and (440) lattice planes of Fe_3_O_4_ NPs (JCPDS No.26‐1136). The diffraction peaks of DMSN‐Au NPs at 2θ angle of 38.1°, 44.4°, 64.5°, and 77.5° appeared, respectively, indexed to the (111), (200), (220), and (311) lattice planes of Au NPs (JCPDS No.04‐0784). The XRD patterns of DMSN‐Fe_3_O_4_ and DMSN‐Au‐Fe_3_O_4_ NPs indicated that the diffraction peaks of Fe_3_O_4_ NPs and Au NPs decreased significantly after encapsulated into the DMSN NPs.

The zeta‐potential variations among DMSN, aminated DMSN (DMSN‐NH_2_), DMSN‐Au, and DMSN‐Au‐Fe_3_O_4_ NPs indicate the desirable synthesis in each step (Figure S8, Supporting Information). Typically, the original DMSN NPs were negatively charged due to the presence of large amounts of silanol groups on surface, and the introduction of surface amine groups by APTES treatment turned the negative (−15.6 mV) zeta potential into positive (+17.4 mV). The highly negative zeta potential (−20.8 mV) of DMSN‐Au‐Fe_3_O_4_ NPs ensures their excellent colloidal stability. The ultraviolet–visible (UV–vis) spectra confirm the successful synthesis of DMSN‐Au NPs and DMSN‐Au‐Fe_3_O_4_ NPs, featuring a typical absorption peak at 505 nm attributed to the plasma resonance effect of Au NPs (Figure S9, Supporting Information). The atomic ratio among Si, Au, and Fe was calculated to be 1:0.67:0.40 by inductively coupled plasma‐optical emission spectrometry (ICP‐OES) measurements.

In tumor regions, owing to the unique GOx‐mimic property of Au nanozyme immobilized within the mesopores of DMSN NPs, DMSN‐Au‐Fe_3_O_4_ NPs initially catalyze glucose oxidation in the presence of oxygen into gluconic acid and H_2_O_2_, which was further catalyzed into highly toxic hydroxyl radicals (·OH) by the coloaded POD‐mimic Fe_3_O_4_ nanozyme via Fenton reaction. In order to evaluate the function of Fe_3_O_4_ nanozyme in producing ·OH, the catalytic performance of DMSN‐Fe_3_O_4_ NPs and the cascade catalytic performance of the biomimetic dual inorganic nanozyme DMSN‐Au‐Fe_3_O_4_ NPs were investigated in detail.

A typical colorimetric method based on 3,3′,5,5′‐tetramethylbenzidine (TMB) was introduced as a substrate to test the POD‐like catalytic activity of DMSN‐Fe_3_O_4_ NPs. In the presence of H_2_O_2_, DMSN‐Fe_3_O_4_ NPs catalyzed the oxidation of TMB to form the oxidized and therefore blue‐colored TMB (oxTMB) featuring characteristic absorbances at 370 and 652 nm (**Figure** 2a). The possible reaction mechanism involves two steps where the O—O bond in H_2_O_2_ molecule was broken into ·OH followed by TMB oxidation by ·OH to form oxTMB. UV–vis absorption spectroscopy was used to monitor the production of the colorimetric product oxTMB. Negligible absorbance can be observed in the absence of DMSN‐Fe_3_O_4_ NPs (Figure 2b), suggesting that no oxidation reaction has occurred in the mixture of TMB and H_2_O_2_. However, an apparently blue‐colored solution can be obtained after the addition of DMSN‐Fe_3_O_4_ NPs into TMB‐H_2_O_2_ mixture solution (pH 6.5) for 10 min with two major absorbance peaks at 370 and 652 nm attributable to oxTMB, which confirms the production of ·OH by DMSN‐Fe_3_O_4_ NPs and H_2_O_2_.

**Figure 2:**
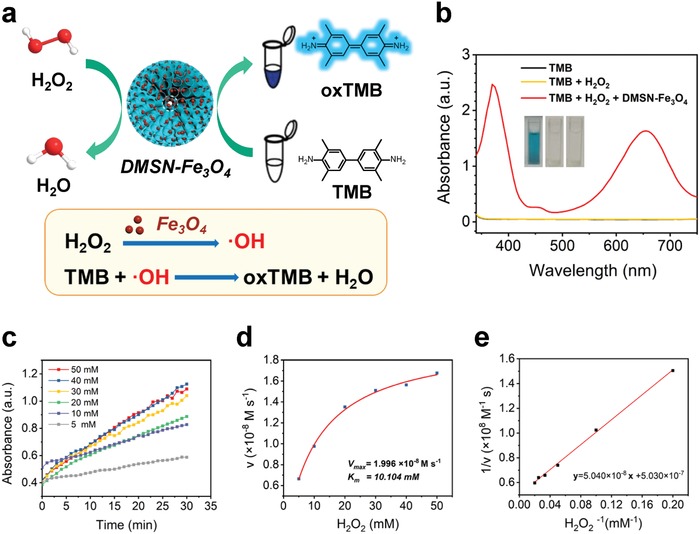
In vitro characterizations of the catalytic performances of DMSN‐Fe_3_O_4_ nanozyme with H_2_O_2_. a) Schematic illustration of the POD‐mimic catalytic process of DMSN‐Fe_3_O_4_ nanozyme. b) UV–vis absorption spectra and visual color changes of the catalyzed oxidation of TMB (oxTMB) as catalyzed by control (black line), H_2_O_2_ (yellow line), and DMSN‐Fe_3_O_4_ + H_2_O_2_ (red line) in the reaction buffer (pH 6.5). Insets show the corresponding digital photos of each group. c) Time‐dependent absorbance changes at 652 nm as a result of the catalyzed oxidation of TMB at different H_2_O_2_ concentrations (5, 10, 20, 30, 40, 50 × 10^−3^ m). d) Michaelis–Menten kinetic analysis and e) Lineweaver–Burk plotting for DMSN‐Fe_3_O_4_ with H_2_O_2_ as substrate. The steady‐state catalytic rate (*v*) was calculated from the initial slopes of absorbance versus time plots in panel (c).

To further evaluate the catalytic activity of DMSN‐Fe_3_O_4_ nanomedicine, the steady‐state catalytic kinetics was investigated at room temperature in a reaction system containing DMSN‐Fe_3_O_4_ NPs, TMB, and H_2_O_2_ of varied concentrations (5, 10, 20, 30, 40, and 50 × 10^−3^ m) in Na_2_HPO_4_‐citric acid buffer solution (pH 6.5). The time‐dependent absorbance variation of the reaction solution was monitored in time‐scan mode at 652 nm using a microplate reader (Figure 2c). At each H_2_O_2_ concentration, the concentration‐changing rate (*v*) of oxTMB was calculated from the absorbance‐changing rate via the Beer–Lambert law, *A* = *εlc* (*A* is the absorbance, ε is the molar absorbance coefficient, *l* is the path length, and *c* is the molar concentration) with *l* = 10 mm and ε of 39 000 m ^−1^ cm^−1^ for oxTMB. Furthermore, the concentration change rates of TMB were plotted against corresponding H_2_O_2_ concentrations, which follows the Michaelis–Menten equation (Figure 2d), known as

(1) $$v_{0}=V_{\max}\left[S\right]/\left(K_{M}+\left[S\right]\right)$$

where *v*_0_ is the initial velocity of the reaction, *V*_max_ is the maximal velocity of reaction, [*S*] is the substrate concentration, and *K*_M_ is the Michaelis–Menten constant. The Michaelis–Menten equation describes the relationship between the rate of substrate conversion and the concentration of the substrate. Generally, the maximal velocity of reaction *V*_max_ reveals the catalytic activity of enzyme. Through an easy deformation, the original Michaelis–Menten equation could be converted to be

(2) $$\;\frac{1}{v_{0}}=\frac{K_{M}}{V_{\max}}\times \frac{1}{\left[S\right]}+\frac{1}{V_{\max}}$$

According to the above equation, *K*_M_ and *V*_max_ of the catalytic reaction by DMSN‐Fe_3_O_4_ NPs were determined by Lineweaver–Burk plot (Figure 2e). The *K*_M_ and *V*_max_ values were calculated to be 10.10 × 10^−3^ m and 1.996 × 10^−8^ m s^−1^ for DMSN‐Fe_3_O_4_ NPs with H_2_O_2_ being the substrate, respectively.

After confirming the catalytic activity of POD‐mimic Fe_3_O_4_ nanozyme in generating highly toxic ·OH from H_2_O_2_ molecules, the catalytic activity of Au NPs as the GOx‐mimic nanozyme and the cascade catalytic performance were investigated. As a GOx mimic, Au NPs catalyze the oxidation of glucose in the presence of oxygen, producing gluconic acid and H_2_O_2_ (**Figure** 3a). Therefore, the production of H_2_O_2_ and gluconic acid, and the consumption of O_2_ were assessed.

**Figure 3:**
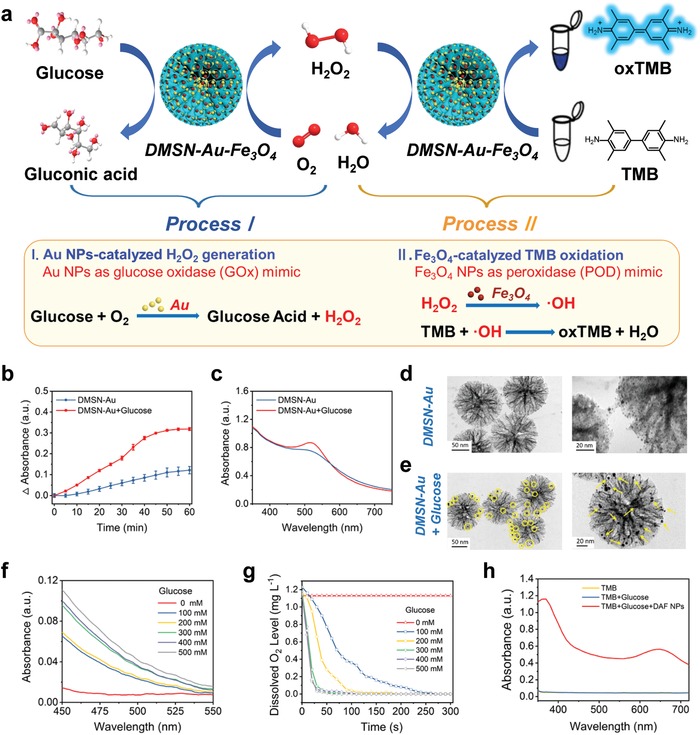
In vitro characterization of the catalytic performances of DMSN‐Au‐Fe_3_O_4_ nanoplatform with glucose. a) Schematic illustration of DMSN‐Au‐Fe_3_O_4_ nanoplatform‐catalyzed glucose oxidation reaction. b) Time‐dependent absorbance changes of DMSN‐Au NPs in the presence or absence of glucose. c) UV–vis absorption spectra of DMSN‐Au NPs in the presence or absence of glucose. TEM images of the enlarged Au NPs in the pore channels of DMSN‐Au NPs under the presence of HAuCl_4_ d) without or e) with the addition of glucose. f) UV–vis absorption spectra of DMSN‐Au NPs after incubation with glucose at varied concentrations (0, 100, 200, 300, 400, 500 × 10^−3^ m) for 3 h. DMSN‐Au NPs‐catalyzed oxidation of glucose to gluconic acid, producing a red complex (505 nm) after mixing with hydroxylamine and FeCl_3_. g) Dissolved oxygen level of DMSN‐Au solution after incubation with glucose at varied concentrations (0, 100, 200, 300, 400, 500 × 10^−3^ m). h) UV–vis absorption spectra of TMB as catalyzed by control (yellow line), glucose (blue line), and DMSN‐Au‐Fe_3_O_4_ NPs + glucose (red line) in the reaction buffer (pH 6.5).

First, the reaction product H_2_O_2_ generated from glucose catalyzed by Au NPs was qualitatively detected based on the H_2_O_2_‐mediated size‐enlargement of Au NPs in the presence of HAuCl_4_.^30^ DMSN‐Au NPs first catalyze the glucose oxidation reaction, and the in situ generated H_2_O_2_ reduces HAuCl_4_ to Au^0^ following the equation

(3) $${\mathrm{AuCl}}_{4}{}^{-}\;\;+3/2\;H_{2}O_{2}\to {\mathrm{Au}}^{0}+4{\mathrm{Cl}}^{-}+3H^{+}+3/2O_{2}$$

The resulting Au species can further deposit on the surface of the initial Au NPs as seeds, causing the enlarged size of initially deposited Au NPs and correspondingly enhanced UV–vis absorbance intensity. According to the UV–vis results, in the presence of glucose and HAuCl_4_, the absorption of Au NPs at 505 nm increased over time (Figure 3b) and the absorption peak slightly red‐shifted from 505 to 515 nm in 1 h of reaction (Figure 3c), suggesting the size‐enlargement of Au NPs. TEM images clearly demonstrate the almost unchanged sizes of Au NPs without glucose addition (Figure 3d) while the significant size enlargement of Au NPs in the presence of glucose (Figure 3e), which can be well‐contributed to the produced H_2_O_2_ via the catalytic glucose oxidation by DMSN‐Au NPs.

The other product of glucose oxidation reaction, gluconic acid, was detected using a gluconic acid‐specific colorimetric assay based on the reaction between gluconic acid, hydroxylamine, and FeCl_3_, which leads to the formation of a red compound hydroxamate‐Fe^3+^ with a typical absorbance peak at 505 nm.^31^ Specifically, DMSN‐Au NPs were incubated with glucose of different concentrations at pH 6.5 for 30 min. After the addition of hydroxylamine and Fe^3+^, the resulting solution turns into red with the major absorbance peak of the reaction solution being at 505 nm and increases with the elevated concentrations of glucose (Figure 3f), which confirms the production of gluconic acid in this Au NPs‐catalyzed reaction and implies the catalytic activity of DMSN‐Au NPs on glucose oxidation.

In order to monitor the level of dissolved oxygen in the reaction system, a dissolved oxygen meter was used. After adding DMSN‐Au NPs into the buffer solution of glucose of different concentrations, the oxygen level in reaction solution declined rapidly owing to the consumption of dissolved oxygen for the glucose oxidation as catalyzed by the loaded Au NPs (Figure 3g).

Based on TMB colorimetric assay (Figure 3a), it has been verified that the self‐organized enzymatic cascade reaction can be achieved by DMSN‐Au‐Fe_3_O_4_ NPs without the aid of any natural enzyme. In detail, DMSN‐Au‐Fe_3_O_4_ NPs and glucose solution were mixed and incubated in a TMB solution for 1 h. The reaction solution then turned blue with the absorbance peak at 370 and 652 nm, indicating the production of ·OH originating from the cascade catalysis of Au and Fe_3_O_4_ NPs (Figure 3h). To be specific, the Au NPs immobilized within the mesopores of DMSN NPs initially catalyze the oxidation of glucose into H_2_O_2_, which is further utilized as the reactant for Fe_3_O_4_ NPs‐based catalytic Fenton reaction to produce hydroxyl radicals. Finally, the generated hydroxyl radicals oxidize the colorless TMB into the blue‐colored oxTMB. Therefore, DMSN‐Au‐Fe_3_O_4_ NP is indeed a robust composite nanozyme with dual enzymatic functionalities capable of catalyzing the cascade reaction without the assistance of any natural enzyme, i.e., initially catalyzing glucose oxidation to yield gluconic acid and H_2_O_2_, and then catalyzing H_2_O_2_ into high‐toxic ·OH. Especially, the catalytic performances of DMSN‐Au‐Fe_3_O_4_ NPs in buffer solutions at pH 5.0, 6.5, 7.4, and 8.0 were conducted to investigate the influence of pH. It has been found that the catalytic activity of DMSN‐Au‐Fe_3_O_4_ NPs is pH‐dependent, exhibiting better catalytic activity in acid condition than in neutral or alkaline condition (Figure S10, Supporting Information).

Before assessing the in vitro nanocatalytic therapeutic efficiency, the surface of DMSN‐Au‐Fe_3_O_4_ NPs was modified with methoxy PEG‐thiol (mPEG‐SH) molecules for improved stability in physiological microenvironment. The Fourier transform infrared spectroscopy (FTIR) was conducted for the characterization of PEGylation. The FTIR spectrum of DMSN‐Au‐Fe_3_O_4_‐PEG NPs shows the stretch of the C—O—C band at 1060, 1110, 1150, 1250, and 1280 cm^−1^, as well as the stretch band of CH_2_ and CH_3_ at 2890 and 2740 cm^−1^, respectively, indicating the successful modification of mPEG‐SH molecules (Figure S11, Supporting Information). Attributing to the PEG modification, the DMSN‐Au‐Fe_3_O_4_‐PEG NPs could be well dispersed in water, phosphate buffer solution, simulated body buffer, saline, dulbecco's modified eagle medium (DMEM), and fetal bovine serum, indicating their excellent stability (Figure S12, Supporting Information).

Initially, the cytotoxicities of DMSN‐Au‐Fe_3_O_4_ NPs were tested on murine breast cancer 4T1 cells, as well as two normal cell lines, namely brain capillary endothelial cells and human umbilical vein endothelial cells by the standard cell‐counting kit 8 assay. The results show that the DMSN‐Au‐Fe_3_O_4_ NPs exhibit negligible effects on the proliferation of the two kinds of normal cells, at the elevated concentration up to 200 µg mL^−1^ within 12 and 24 h, indicating their high biosafety and biocompatibility for further in vitro and in vivo therapeutic applications (Figure S13, Supporting Information). In addition, it has been found that DMSN‐Au NPs and DMSN‐Fe_3_O_4_ NPs exhibit no significant inhibition on 4T1 cancer cells proliferation even at the elevated concentration up to 200 µg mL^−1^ after incubation for 12 and 24 h, at pH 6.5 (**Figure** 4a,b) and 7.4 (Figure S14, Supporting Information). Importantly, when incubated with DMSN‐Au‐Fe_3_O_4_ NPs, the cell viabilities exhibit a marked decline in a dose‐dependent manner with an inhibition rate on 4T1 tumor cell viability being higher than 75% at 200 µg mL^−1^ (Figure 4c). This result indicates that the cytotoxicity of DMSN‐Au‐Fe_3_O_4_ NPs should be attributed to the cascade catalytic reactions by Au and Fe_3_O_4_ NPs, both of which are indispensable to trigger the generation of sufficiently high amount of toxic ·OH to induce the apoptosis of 4T1 tumor cells. In detail on the related therapeutic mechanism, H_2_O_2_, as produced by the reaction between glucose and oxygen under the catalysis by GOx mimic‐Au NPs in cell culture medium, is further catalyzed by Fe_3_O_4_‐based Fenton nanocatalysts to disproportionate, producing ·OH for killing the cancer cells.

**Figure 4:**
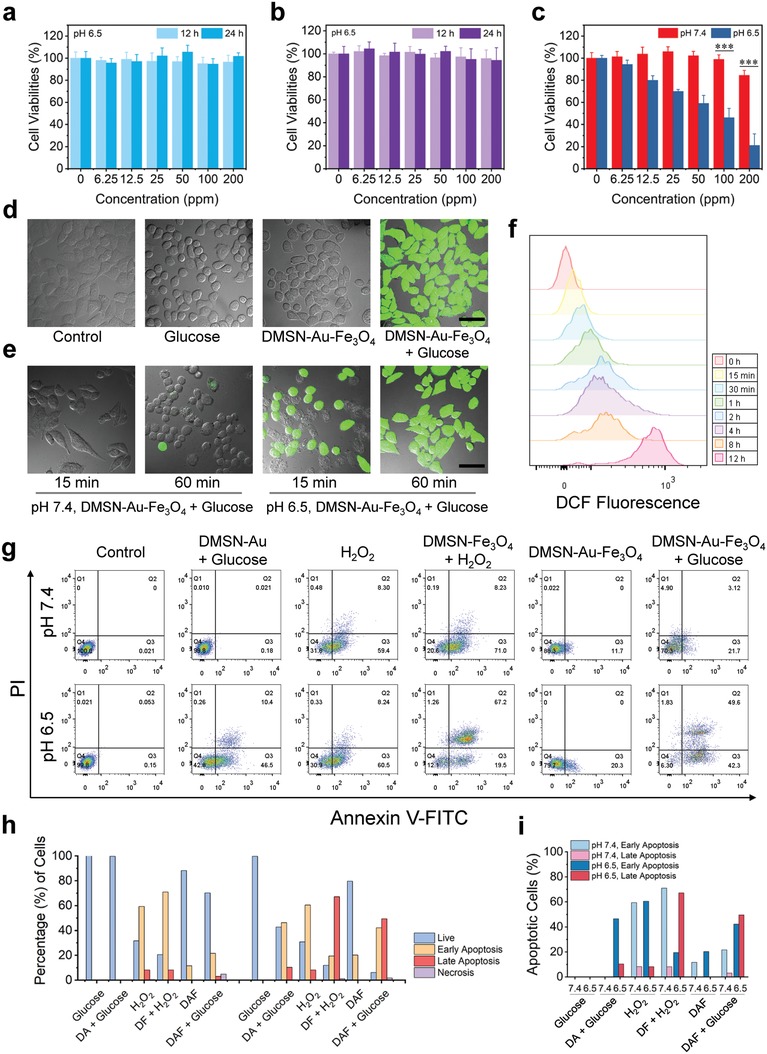
In vitro assessments of the nanocatalytic tumor therapeutic efficacy by DMSN‐Au‐Fe_3_O_4_ nanoplatform. Relative viabilities of 4T1 cells after being incubated with a) DMSN‐Au NPs and b) DMSN‐Fe_3_O_4_ NPs at varied concentrations (0, 6.25, 12.5, 25, 50, 100, and 200 µg mL^−1^) for 12 and 24 h at pH 6.5. c) Relative viabilities of 4T1 cells after being incubated with DMSN‐Au‐Fe_3_O_4_ NPs at varied concentrations (0, 6.25, 12.5, 25, 50, 100, and 200 µg mL^−1^) for 24 h at pH 7.4 and pH 6.5 (*P* values: ***P <0.001). d) Confocal fluorescence imaging of DCFH‐DA stained 4T1 cells after various treatments (Control, Glucose, DMSN‐Au‐Fe_3_O_4_, DMSN‐Au‐Fe_3_O_4_ + Glucose) for 1 h at pH 6.5. Scale bar: 50 µm. e) Confocal fluorescence imaging of DCFH‐DA stained 4T1 cells after incubated with DMSN‐Au‐Fe_3_O_4_ NPs at pH 7.4 and 6.5 for 15 min and 1 h. Scale bar: 50 µm. f) Flow cytometric analysis of intracellular ROS levels in 4T1 cells incubated with DMSN‐Au‐Fe_3_O_4_ NPs for different durations of 0 min, 15 min, 30 min, 1 h, 2 h, 4 h, 8 h, and 12 h. g) Flow cytometric apoptosis analysis of Annexin V‐FITC/PI stained 4T1 cells after different treatments at pH 7.4 and 6.5 for 4 h: Control (Glucose), DMSN‐Au + Glucose, H_2_O_2_, DMSN‐Fe_3_O_4_ + H_2_O_2_, DMSN‐Au‐Fe_3_O_4_, DMSN‐Au‐Fe_3_O_4_ + Glucose. h) Quantification analysis of the percentages of live, early apoptotic, late apoptotic and necrotic cells after different treatments in panel (g). i) Quantification analysis of the percentages of apoptotic cells at pH 7.4 and pH 6.5 after different treatments.

Furthermore, the intracellular uptake of fluorescein isothiocyanate (FITC)‐labeled DMSN‐Au‐Fe_3_O_4_ NPs was investigated by flow cytometry and confocal laser scanning microscopy (CLSM). The flow cytometric results reveal a substantial enhancement of fluorescence intensity of FITC‐labeled DMSN‐Au‐Fe_3_O_4_ NPs at the prolonged incubation duration (Figure S15, Supporting Information). CLSM images of 4T1 tumor cells clearly exhibit the green fluorescence originated from FITC‐labeled DMSN‐Au‐Fe_3_O_4_ NPs after coincubation for 2 h, indicating the efficient intracellular uptake of DMSN‐Au‐Fe_3_O_4_ NPs (Figure S16, Supporting Information) and guaranteeing the following efficient intracellular cascade nanocatalytic reactions for killing the cancer cells.

To evaluate the intracellular ·OH generation, a ROS fluorescence probe 2′,7′‐dichlorofluorescin diacetate (DCFH‐DA), which turns into 2′,7′‐dichlorofluorescein (DCF) with strong green fluorescence in the presence of ROS, was introduced to stain cancer cells in order to reveal the intracellular ROS production. At pH 6.5, 4T1 cancer cells exhibit negligible fluorescence when coincubated only with high doses of glucose in DMEM or with DMSN‐Au‐Fe_3_O_4_ NPs in glucose‐free DMEM for 1 h. Comparatively, strong green fluorescence of 4T1 cancer cells can be observed after coincubation with DMSN‐Au‐Fe_3_O_4_ NPs in the presence of glucose (Figure 4d), implying the massive intracellular ROS production by DMSN‐Au‐Fe_3_O_4_ NPs and glucose under weakly acidic condition. Furthermore, it has been found that the DCF fluorescence intensity is dependent on the pH value of cell culture medium and incubation duration. As the incubation duration is prolonged from 15 min to 1 h, the DCF fluorescence intensity of cells increases accordingly when incubated with DMSN‐Au‐Fe_3_O_4_ NPs and glucose under the weakly acidic condition (pH 6.5). However, in the neutral condition (pH 7.4), much weaker DCF fluorescence intensity is observed even after prolonged incubation (Figure 4e). The DCF fluorescence can be further monitored by flow cytometry (Figure 4f), which demonstrates that the DCF fluorescence increases by prolonging the incubation duration of DMSN‐Au‐Fe_3_O_4_ NPs. The CLSM and flow cytometry results demonstrate that the intracellular ROS production as catalyzed by DMSN‐Au‐Fe_3_O_4_ NPs has been substantially promoted in the mildly acidic environment as compared to that in the neutral condition.

Especially, the intracellular nanocatalytic therapeutic mechanism of DMSN‐Au‐Fe_3_O_4_ nanoplatform was investigated by the typical flow cytometric apoptosis assay after stained with FITC‐labeled Annexin V and propidium iodide (PI). According to the fluorescence intensity, cell populations are assigned into four quadrants including live, early apoptotic, late apoptotic, and necrotic cells (Figure 4g). 4T1 cells were incubated in different conditions including Control (Glucose), DMSN‐Au + Glucose, H_2_O_2_, DMSN‐Fe_3_O_4_ + H_2_O_2_, DMSN‐Au‐Fe_3_O_4_, and DMSN‐Au‐Fe_3_O_4_ + Glucose at pH 7.4 or pH 6.5. The quantitative analysis on the cell populations in four quadrants in each group was also conducted (Figure 4h). At pH 7.4, compared to the control group, cells incubated with H_2_O_2_ group exhibit apparent apoptosis owing to the cytotoxicity of the moderate‐toxic ROS, H_2_O_2_. The cells treated with DMSN‐Fe_3_O_4_ + H_2_O_2_ show a significantly promoted apoptosis, indicating the extra production of highly toxic ROS, hydroxyl radicals. In comparison, cells in group of DMSN‐Au + Glucose, DMSN‐Au‐Fe_3_O_4_, and DMSN‐Au‐Fe_3_O_4_ + Glucose display negligible apoptosis at pH 7.4, indicating that neither DMSN‐Au NPs nor DMSN‐Au‐Fe_3_O_4_ NPs contribute to the ROS production and cell apoptosis in the netural environment. However, at pH 6.5, cells in group of DMSN‐Fe_3_O_4_ + H_2_O_2_ show enhanced late apoptosis compared to those at pH 7.4, which demonstrate that the acidity‐benefited Fenton reaction catalyzed by Fe_3_O_4_ nanozyme has produced high‐toxic hydroxyl radicals for inducing the cell apoptosis. More importantly, cells in group of DMSN‐Au‐Fe_3_O_4_ + Glucose exhibit the mostly enhanced cell apoptosis rate up to about 90% at pH 6.5 while only a slight early apoptosis rate at pH 7.4 is detectable, suggesting the massive ROS production and cell apoptosis as contributed by the cascade catalytic reaction of Au NPs and Fe_3_O_4_ NPs under the mildly acidic condition (Figure 4i). The flow cytometric results indicate the much enhanced efficiency of ROS production and cell apoptosis of nanocatalytic therapy as triggered by biomimetic DMSN‐Au‐Fe_3_O_4_ nanomedicine via cascade catalytic reactions.

The in vivo toxicity assessment of DMSN‐Au‐Fe_3_O_4_ composite nanocatalysts has demonstrated the high biosafety and biocompatibility of DMSN‐Au‐Fe_3_O_4_ NPs for further in vivo therapeutic application on combating cancer (Figures S17 and S18 and Discussion S1, Supporting Information). Furthermore, the blood circulation and biodistribution of DMSN‐Au‐Fe_3_O_4_ NPs were comprehensively investigated. The circulation half‐life of DMSN‐Au‐Fe_3_O_4_ NPs in the bloodstream was calculated to be 1.38 h based on a two‐compartment pharmacokinetic model (**Figure** 5a).^31^ Importantly, after i.v. administration, DMSN‐Au‐Fe_3_O_4_ NPs efficiently accumulated into the tumor tissue with the passive accumulation efficacy of 3.49% ID g^−1^ in 2 h and 1.80% ID g^−1^ in 12 h (Figure 5b) based on the typical EPR effect for tumor‐passive targeting and accumulation.^32^

**Figure 5:**
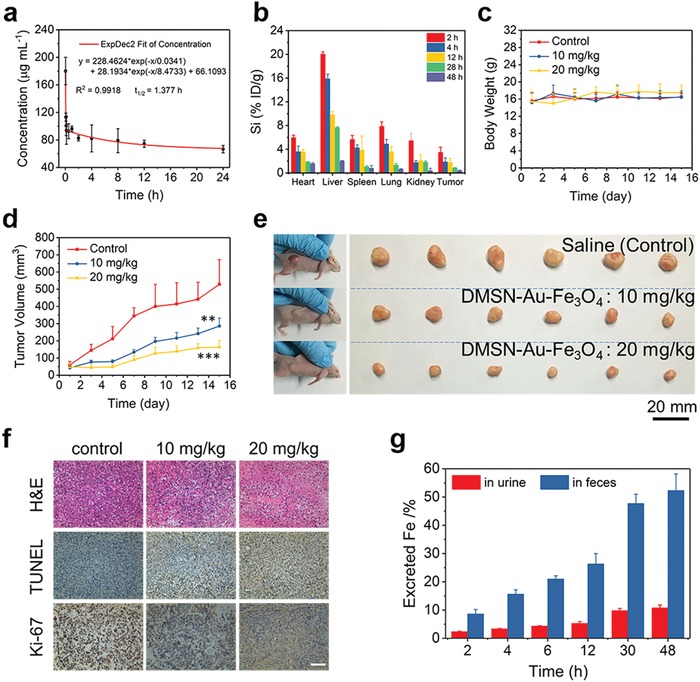
In vivo pharmacokinetic analysis and nanocatalytic tumor therapy. a) Blood‐circulation curve of intravenously injected DMSN‐Au‐Fe_3_O_4_ NPs after i.v. injection (*n* = 3). The half‐life (*T*_1/2_) was calculated to be ≈1.38 h. b) Biodistribution of Si element after i.v. injection of DMSN‐Au‐Fe_3_O_4_ NPs (dose: 20 mg kg^−1^) in 4T1 tumor‐bearing mice measured at different time points as determined by ICP‐OES. c) Time‐dependent body‐weight curves of 4T1 tumor‐bearing nude mice after i.v. injection with saline (control) and DMSN‐Au‐Fe_3_O_4_ NPs (10 and 20 mg kg^−1^). d) Time‐dependent tumor growth curves (*n* = 6, mean ± s.d.) of mice from each group (*P* values: **P <0.01, ***P <0.001). e) Digital photos of 4T1 tumor‐bearing mice and dissected tumors from each group on the 15th day. f) H&E staining, TUNEL staining for pathological changes, and Ki‐67 immunohistochemical staining for cellular proliferation in tumor tissues from each group after a 15‐day therapeutic period. Scale bar: 100 µm. g) Accumulated Fe (in urine and feces) excretion out of the mice body (*n* = 3) after i.v. injection of DMSN‐Au‐Fe_3_O_4_ NPs (dose: 20 mg kg^−1^) for different durations (2, 4, 6, 12, 30, and 48 h).

Encouraged by the high efficiency of in vitro nanocatalytic therapy for killing cancer cells, the prolonged blood circulation, and potent tumor accumulation effect, the in vivo nanocatalytic therapeutic efficiency of DMSN‐Au‐Fe_3_O_4_ NPs was evaluated against the 4T1 breast tumor xenograft on nude mice. Eighteen 4T1 tumor‐bearing mice (tumor volume ≈ 50 mm^3^) were randomly separated into three groups (*n* = 6 per group). Saline (control) and DMSN‐Au‐Fe_3_O_4_ NPs at different doses (10 and 20 mg kg^−1^) were i.v. administrated to investigate the therapeutic performance and related in vivo mechanism. During a 15 day therapeutic period, the body weights of mice in two therapeutic groups show no significant difference from those of mice in the control group (Figure 5c). The tumor volumes of mice in each group were recorded using a digital caliper, and the digital photos of mice were taken every 2 days after the i.v. injection(Figure S19, Supporting Information). It has been found that the i.v. administration of DMSN‐Au‐Fe_3_O_4_ NPs shows a dose‐dependent tumor‐growth inhibition effect during the therapeutic period (Figure 5d,e), with the inhibition rates of 45.96% and 69.08% at the doses of 10 and 20 mg kg^−1^, respectively. This substantial tumor inhibition effect is attributed to the high‐toxic hydroxyl radicals as produced by the endogenous cascade reaction triggered by the biomimetic Au and Fe_3_O_4_ coloaded nanoplatforms under the mildly acidic TME, effectively inducing tumor cell death. It should be noted that the constructed nanocomposites are highly biocompatible without any toxic substance used, and the toxic effect can only be triggered under the mildly acidic TME, which means that this DMSN‐Au‐Fe_3_O_4_‐based nanocatalytic tumor therapy features high tumor specificity and excellent therapeutic biosafety. The neutral condition of normal tissue will not trigger such a cascade catalytic reaction, therefore no toxic effect will be induced to cause noticeable damages to normal cells/tissues.

In order to reveal the detailed therapeutic mechanism by tumor‐pathological analysis, hematoxylin and eosin (H&E), terminal deoxynucleotidyl transferase mediated dUTP nick‐end labeling (TUNEL) and Ki‐67 antibody staining of dissected tumor tissues from each group were conducted. H&E and TUNEL staining results exhibit severe damage and necrosis of tumor cells in two therapeutic groups in comparison to the control group (Figure 5f). Ki‐67 antibody staining results reveal the suppressed proliferative activities of tumors cells in two therapeutic groups while there is almost no significant adverse effect on cell proliferation in the control group. Furthermore, H&E staining of the major organs (heart, liver, spleen, lung, and kidney) conducted after the therapeutic period shows no noticeable pathological side effects on major organs of mice in therapeutic groups (Figure S20, Supporting Information), indicating the high biocompatibility and therapeutic biosafety of DMSN‐Au‐Fe_3_O_4_ NPs as nanocatalytic agents.

Additionally, the rapid clearance of therapeutic nanomaterials is highly favorable for the clinical translation, which can avoid long‐term body retention and potential toxicity. To investigate the metabolism process, 4T1‐tumor‐bearing mice were intravenously injected with DMSN‐Au‐Fe_3_O_4_ NPs (20 mg kg^−1^, *n* = 3) and their urine and feces were collected at predetermined time points (2, 4, 6, 12, 30, and 48 h) after the injections. The Fe element levels in urine and feces were quantitatively determined by ICP‐OES. The excretion pathways including urine and feces reveal the gradual excretion of DMSN‐Au‐Fe_3_O_4_ NPs out of the body with nearly 70% of Fe being excreted out of mice bodies in 48 h post i.v. injection (Figure 5g). The desirable excretion behavior of DMSN‐Au‐Fe_3_O_4_ NPs indicates their potential biocompatibility and biosafety as nanocatalytic agents for further clinical translation.

In summary, this work reports on the effective TME‐responsive catalytic cascade reactions for nanocatalytic tumor‐specific therapy with marked therapeutic efficacy and biosafety based on an inorganic biomimetic DMSN‐Au‐Fe_3_O_4_ composite nanoplatforms. The in situ grown Au NPs within the large mesopores of DMSN NPs as a GOx‐mimic nanozyme specifically catalyze β‐D‐glucose oxidation into gluconic acid and H_2_O_2_ under aerobic conditions, while the produced H_2_O_2_ can subsequently be catalyzed by the coloaded Fe_3_O_4_‐based Fenton nanocatalysts to produce high‐toxic hydroxyl radicals which substantially induce tumor‐cell death afterward. Both the comprehensive in vitro cell‐level and in vivo tumor‐bearing mice xenograft evaluations have demonstrated the efficient nanocatalytic tumor therapy on killing the cancer cells and suppressing the tumor growth by as high as 69.08% of inhibition rate without any toxic substance being used, in highly TME‐responsive and tumor‐specific manners. The high biocompatibility, high therapeutic biosafety, and easy excretion of these DMSN‐Au‐Fe_3_O_4_ composite nanoplatforms have also been verified which guarantees their further clinical translation. Therefore, the present biomimetic dual inorganic nanocomposite‐triggered cascade reaction strategy for TME‐responsive and effective nanocatalytic tumor therapy not only establishes a paradigm of “toxic‐drug‐free” endogenous and noninvasive nanocatalytic biomedicine, but also takes an important step forward in developing biomimetic nanoplatforms with multienzyme mimicking catalytic activities for tumor‐specific therapies.

## Conflict of Interest

The authors declare no conflict of interest.

## Supporting information

Supplementary
