# Supplementary material for: Nanocatalytic Tumor Therapy by Biomimetic Dual Inorganic Nanozyme‐Catalyzed Cascade Reaction
Source: Adv Sci (Weinh). 2018 Nov 12;6(3):1801733. doi: 10.1002/advs.201801733 (PMC6364502; doi:10.1002/advs.201801733)
Supplement: Supplementary file 1 — Supplementary [file ADVS-6-1801733-s001.pdf]

Copyright WILEY-VCH Verlag GmbH & Co. KGaA, 69469 Weinheim, Germany, 2018.

ADVANCED SCIENCE
Open Access

Supporting Information

for *Adv. Sci.,* DOI: 10.1002/advs.201801733

Nanocatalytic Tumor Therapy by Biomimetic Dual Inorganic Nanozyme-Catalyzed Cascade Reaction

Shanshan Gao, Han Lin, Haixian Zhang, Heliang Yao, Yu Chen, and Jianlin Shi**

Supporting Information

Nanocatalytic Tumor Therapy by Biomimetic Dual Inorganic Nanozyme-catalyzed Cascade Reaction

Shanshan Gao, Han Lin, Haixian Zhang, Heliang Yao, Yu Chen* and Jianlin Shi*

Miss S. Gao, Mr. H. Lin, Mr. H. Yao, Prof. Y. Chen, Prof. J. Shi
State Key Laboratory of High Performance Ceramics and Superfine Microstructure, Shanghai Institute of Ceramics, Chinese Academy of Sciences, Shanghai 200050, P. R. China.
E-mail: chenyu@mail.sic.ac.cn (Y. Chen); jlshi@mail.sic.ac.cn (J. Shi).

Miss S. Gao, Mr. H. Lin
University of Chinese Academy of Sciences, Beijing 100049, P. R. China.

Dr. H. Zhang
Department of Ultrasound, Fudan University Shanghai Cancer Center; Department of Oncology, Shanghai Medical College, Fudan University, Shanghai 200032, P. R. China.

A: Experimental Section

1. Materials and Reagents

Tetraethyl orthosilicate (TEOS), hydrochloric acid (HCl), acetone, ammonia solution (30%), and ethanol were obtained from Sinopharm Chemical Reagent Co., Ltd (Shanghai, China). Cetyltrimethylammonium bromide (CTAB), sodium salicylate (NaSal), triethanolamine (TEA), sodium borohydride ($NaBH_4$), β-D-glucose, H_2O_2, $HAuCl_4 \cdot 4H_2O$, fluorescein isothiocyanate (FITC), 2,7-dichlorofluorescin diacetate (DCFH-DA) and 3,3’,5,5’-tetramethylbenzidine (TMB) were purchased from Sigma-Aldrich Co., Ltd (USA). Iron chloride ($FeCl_3 \cdot 6H_2O$), oleyl alcohol, sodium oleate, chloroform, diphenyl ether, sodium phosphate dibasic (Na_2HPO_4), citric acid (CA) and n-hexane were obtained from Shanghai Macklin Biochemical Co., Ltd. Methoxy PEG-thiol (mPEG-SH, Mw = 5000) was ordered

from Shanghai Yare Biotech, Co., Ltd. All chemicals were used as received without further purification.

2. Synthesis of Dendritic Mesoporous Silica Nanoparticles (DMSN NPs)

DMSN NPs were synthesized according to the previous report.[1] Briefly, TEA (0.068 g) was diluted in pure water (25 mL) at 80 °C under magnetic stirring for 0.5 h, followed by adding NaSal (168 mg) and CTAB (380 mg) to the reaction solution and keeping stirring for another 1 h. Then, TEOS (4 mL) was added dropwise into the reaction solution with gentle stirring (~ 300 rpm) for 2 h. The products were collected by high-speed centrifugation (20000 rpm, 15 min) and washed several times with water and ethanol to remove the residual reactants. Finally, the obtained products were extracted with HCl and ethanol solution (HCl: ethanol = 1:9) at 80.0 °C (6 h, three times) to remove the surfactants (CTAB) and dried in vacuum at room temperature overnight. The size of DMSN NPs could be easily adjusted from 130 nm to 320 nm by changing the CTAB/NaSal molar ratio from 1/0.25 to 1/1.5 (**Figure S1**).

3. Synthesis of 1.5 nm-sized Ultra-small Fe_3O_4 NPs

Uniform 1.5 nm-sized iron oxide nanoparticles (Fe_3O_4 NPs) were synthesized by thermal decomposition of iron-oleate complex.[2] Initially, iron-oleate complex was prepared through the reaction of $FeCl_3$ and sodium oleate. Briefly, $FeCl_3 \cdot 6H_2O$ (2.7 g) and sodium oleate (9.125 g) were dissolved in the mixture solvent of ethanol (20 mL), pure water (15 mL) and n-hexane (35 mL). The above solution was heated to 70 °C and kept for 4 h. Then, the upper organic layer was washed with distilled water three times in a separatory funnel followed by rotary evaporation to obtain the waxy solid, iron-oleate complex. After that, the as-prepared iron-oleate complex (9 g) and oleyl alcohol (16.1 g) were dissolved in diphenyl ether (50 g). The mixture solution was heated to 200 °C at a constant heating rate of 10 °C/min and kept for 30 min under inert atmosphere. Then the reaction solution was swiftly cooled to room temperature, followed by the addition of acetone (100 mL) to precipitate the Fe_3O_4 NPs. The

final monodispersed 1.5 nm-sized Fe_3O_4 NPs were obtained by centrifugation and dispersed in chloroform for further use.

4. Synthesis of DMSN-Au NPs

DMSN NPs (100 mg) were dispersed into deionized water (10 mL), followed by the addition of $HAuCl_4$ solution (1 mL, 20 mM). After sonication for 20 min, the freshly prepared $NaBH_4$ solution (5 mL, 0.1 M) was quickly added into the above solution under vigorous stirring. The resulting DMSN-Au NPs were obtained by centrifugation after stirring for 1 h.

5. Synthesis of DMSN-Au-Fe_3O_4 NPs and DMSN-Fe_3O_4 NPs

DMSN-Au NPs (20 mg) were dispersed in n-hexane(10 mL) by sonication, followed by adding Fe_3O_4 n-hexane solution (1 mL, 5 mg mL^{-1}). After stirring for 12 h, the DMSN-Au-Fe_3O_4 NPs were obtained by centrifugation. DMSN-Fe_3O_4 NPs were prepared similarly by loading Fe_3O_4 NPs into the DMSN NPs.

6. PEGylation of DMSN-Au-Fe_3O_4 NPs

DMSN-Au-Fe_3O_4 NPs (10 mg) and mPEG-SH (5 mg) were dispersed in anhydrous ethanol (10 mL) and stirred for 12 h. After collected by centrifugation and washed with water and ethanol, the PEGylated DMSN-Au-Fe_3O_4 NPs were obtained.

DMSN-Au-Fe_3O_4 NPs and DMSN-Au-Fe_3O_4-PEG NPs (10 μg mL^{-1}) in diverse solutions ware rested for 30 min and 2 h to evaluated the dispersing stability of DMSN-Au-Fe_3O_4 NPs after PEGylation.

7. Catalytic Activities of DMSN-Fe_3O_4 and DMSN-Au-Fe_3O_4 NPs

TMB was applied as substrates to investigate the catalytic activity of DMSN-Fe_3O_4 and DMSN-Au-Fe_3O_4 NPs. Na_2HPO_4-citric acid buffer solution (pH 3-8) was prepared as the reaction buffer. The steady-state kinetic catalytic activities of DMSN-Fe_3O_4 and DMSN-Au-Fe_3O_4 NPs were investigated by monitoring the absorbance variation at 652 nm of the reaction buffer using a microplate reader (SpectraMax M2, Molecular Devices, USA). The

Michaelis-Menten kinetics model was applied to analyze the kinetic catalytic behaviors of DMSN-Fe_3O_4 NPs.

8. Material Characterization

Transmission electron microscopy (TEM) images were acquired on a JEOL-2100F transmission electron microscope. Scanning electron microscopy (SEM) images and corresponding element mapping scanning were acquired on a field-emission Magellan 400 microscope (FEI Company, USA). The nitrogen (N_2) adsorption-desorption isothermal curve and corresponding pore-size distribution of DMSN NPs were measured by a Micromeritics Tristar 3000 system. The quantitative elemental analysis was determined by inductively coupled plasma-optical emission spectrometry (ICP-OES, Agilent 725, Agilent Technologies, USA). Zeta potential measurement was recorded on a Zetasizer Nanoseries (Nano ZS90, Malvern Instrument Ltd.). Fourier transform infrared spectroscopy (FT-IR) spectra were acquired by a Thermo Scientific Nicolet iS 10 FT-IR spectrometer. X-ray diffraction (XRD) experiment was conducted on a Rigaku D/MAX-2200 PC XRD system at Cu K_{α} (λ = 0.154056 nm). UV-vis absorption spectra were recorded with a UV-3600 Shimadzu UV-vis spectrometer. Confocal laser scanning microscopy (CLSM) images were acquired by FV1000 (Olympus Company, Japan). Intracellular uptake and cell apoptosis were obtained by a BD LSRFortessa flow cytometry (Becton, Dickinson and Company, USA).

9. Cell Culture

Murine breast cancer 4T1 cell line (4T1 cells), brain capillary endothelial (BCEC) cells and human umbilical vein endothelial (HUVEC) cells were obtained from Cell Bank of Shanghai Institutes for Biological Sciences, Chinese Academy of Sciences and were cultured in a humidified incubator (Thermo Fisher Scientific Inc. USA) with 5% CO_2 at 37°C in Dulbecco's Modified Eagle Medium (DMEM, GIBCO, Invitrogen) supplemented with 10% fetal bovine serum (FBS), penicillin (100 units mL^{-1}) and streptomycin (100 mg mL^{-1}).

10. In vitro Cytotoxicity Measurements and Apoptosis Analysis

To evaluate the cytotoxicity of DMSN-Au-Fe_3O_4 NPs, a standard Cell Counting Kit-8 (CCK-8) assay (Sihai Bio-Tech Co., Ltd, Shanghai) was conducted. 4T1 cancer cells, BCEC cells and HUVEC cells were seeded (1×10^4 cells in 100 μL of DMEM per well) in sextuplicate in 96-well microplates and allowed to adhere overnight. To simulate the acidic (pH 6.5-6.9) extracellular microenvironment in solid tumor, DMEM (pH 7.4) was acidized to pH 6.5 by hydrochloric acid. Then the culture media were replaced by fresh medium (pH 7.4 or 6.5) containing DMSN-Au-Fe_3O_4 NPs at varied concentrations of 6.25, 12.5, 25, 50, 100, 200 μg mL^{-1}. After further incubation for 12 h or 24 h, the culture media were replaced by FBS-free medium containing 10% CCK-8. After further co-incubation for 1 h, the cell proliferation was determined using the microplate reader by comparing the absorbance at 450 nm to the control group. The cytotoxicities of DMSN-Au NPs (6.25, 12.5, 25, 50, 100, 200 μg mL^{-1}), DMSN-Fe_3O_4 NPs (6.25, 12.5, 25, 50, 100, 200 μg mL^{-1}), and H_2O_2 (0, 50, 100, 200, 300, 400, 500 and 1000 μM) to 4T1 cells after incubation for 12 h and 24 h were tested similarly as mentioned above.

Flow cytometry was conducted for cell apoptosis analysis. 4T1 cells were seeded (1×10^5 cells in 1 mL per well) in 6-well plates and allowed to adhere overnight. Then the culture media were replaced by DMSN-Au-Fe_3O_4 NPs (1 mL, 50 μg mL^{-1}) in DMEM (pH 7.4 or 6.5) and incubated for another 6 h. After co-incubation, the cells were collected by centrifugation. Then, the Annexin V-FITC/PI Apoptosis Detection Kit (Dojindo Molecular Technologies, Inc., Japan) was used to stain the cells for 20 min before the flow cytometry analysis.

11. Intracellular Endocytosis Analysis

The intracellular endocytosis process of DMSN-Au-Fe_3O_4 NPs was investigated by flow cytometry and confocal laser scanning microscopy. For flow cytometry test, 4T1 cells were seeded (1×10^5 cells/well, pH 7.4) into 6-well plates and allowed to adhere overnight. Then the culture media were replaced by FITC-labeled DMSN-Au-Fe_3O_4 NPs in DMEM (1 mL, 50 μg mL^{-1}). After co-incubation for 0, 15 min, 30 min, 1 h, 2 h, 4 h, 8 h and 12 h, the 4T1 cells were collected by trypsin and transferred into the test tube and flow cytometry analysis was

performed to evaluate the fluorescence intensity of FITC-labeled DMSN-Au-Fe_3O_4 NPs endocytosed in cancer cells.

For CLSM observation, 4T1 cells were seeded (1×10^5 cells in 1 mL DMEM) in the CLSM-exclusive culture dishes and allowed to adhere overnight. Then the culture media were replaced by FITC-labeled DMSN-Au-Fe_3O_4 NPs (1 mL, 50 μg mL^{-1}) in DMEM. After varied co-incubation durations (0, 1, and 2 h), 1 mL 4',6-diamidino-2-phenylindole (DAPI, Beyotime Biotechnology) diluted by methyl alcohol (1:15) was applied to stain cell nuclei for 20 min.

12. Intracellular ROS Evaluation

CLSM was introduced to evaluate the ROS generation. Initially, the glucose-responsive ROS generation of DMSN-Au-Fe_3O_4 NPs at acidic culture environment was evaluated. 4T1 cells were seeded (1×10^5 cells in 1 mL DMEM, pH 6.5) in the CLSM-exclusive culture dish and allowed to adhere overnight. The culture media were then replaced by 1 mL of acidulated DMEM (pH 6.5) as the following groups: DMSN-Au-Fe_3O_4 NPs (50 μg mL^{-1}) in glucose-free DMEM, 4.5 mg mL^{-1} glucose-containing DMEM, and DMSN-Au-Fe_3O_4 NPs (50 μg mL^{-1}) in 4.5 mg mL^{-1} glucose-containing DMEM. After incubation for 1 h, the culture media were replaced by 1 mL DCFH-DA (10 μM in FBS-free DMEM) and incubated for 30 min. The cells were washed with PBS three times and the level of intracellular ROS was evaluated by detecting the fluorescence of DCF (λ_{ex} = 488 nm, λ_{em} = 525 nm). Afterwards, in order to study the influence of pH and incubation duration on the ROS generation of DMSN-Au-Fe_3O_4 NPs, 4T1 cells were seeded in pH 7.4 or pH 6.5 (1×10^5 cells in 1 mL DMEM) in the CLSM-exclusive culture dishes and allowed to adhere overnight. After incubation for 15 min or 1 h, the culture media were replaced by 1 mL DCFH-DA (10 μM in FBS-free DMEM) and incubated for 30 min. The cells were washed with PBS three times and the intracellular ROS was evaluated with CLSM as mentioned above.

13. In Vivo Toxicity Assay

All animal experiments operations were performed in accordance with the protocols approved by Institutional Animal Care and Use Committee (IACUC) and the care regulations approved by the administrative committee of laboratory animals of Fudan University. Twenty SBF-leveled four-week-old healthy female Kunming mice (~ 20 g) were obtained and raised at Laboratory Animal Center, Shanghai Medical College of Fudan University. The mice were randomly divided into four groups (n = 5): (1) control group, mice intravenously administered with DMSN-Au-Fe_3O_4 NPs (100 μL saline, dose: 20 mg kg^{-1}) for (2) 1 d, (3) 7 d and (4) 28 d at the dosage of 20 mg kg^{-1}. Their body weights were measured every two days. Over the 28-day period, no significant behavioral changes were observed after intravenous injection of DMSN-Au-Fe_3O_4 NPs compared to the control group. At the given intervals, histological analysis, hematological and blood biochemical indexes were collected.

14. Pharmacokinetics, Biodistribution and Metabolism Studies

Pharmacokinetic analysis of DMSN-Au-Fe_3O_4 NPs was conducted on healthy female Kunming mice (n = 3). 10 μL of blood was collected at varied time intervals (2 min, 5 min, 8min, 10 min, 15 min, 30 min, 1 h, 2 h, 4 h, 8 h, 12 h and 24 h) after intravenous injection of 100 μL of DMSN-Au-Fe_3O_4 NPs (20 mg kg^{-1}). The quantitative analysis of Si element was determined by ICP-OES. Origin software was used for data analysis for the best-fit line and blood terminal half-life based on the two-compartment pharmacokinetic model.

Biodistribution analysis of DMSN-Au-Fe_3O_4 NPs in tumor tissues and major organs were performed in 4T1 tumor-bearing mice (n = 3). To establish the animal tumor model, 4T1 xenografted tumors were generated in 4-week-old female Balb/c mice by subcutaneously (s.c.) injecting of 4T1 cells (1×10^6 cell/site, 100 μL saline) into the right rear legs of female Balb/c Nude mice. 4T1 tumor-bearing mice were intravenously injected with 100 μL of DMSN-Au-Fe_3O_4 NPs in saline (20 mg kg^{-1}). Mice were sacrificed and dissected at predesignated time intervals (2, 4, 12, 28 and 48 h) to collect the tumors and major organs. These sections were weighed, homogenized and dissolved in aqua regia. The Fe distribution

in different tissues was calculated as the percentage of the original injected dose per gram of tissue.

Metabolism process of DMSN-Au-Fe_3O_4 NPs was investigated on 4T1 tumor-bearing mice (n = 3). DMSN-Au-Fe_3O_4 NPs in saline (20 mg·kg^{-1} per mouse, 100 μL) were intravenously injected into the 4T1 tumor-bearing mice. The urine and feces were collected at different time intervals (2, 4, 6, 12, 30 and 48 h). The Fe contents in urine and feces ware quantitatively determined by ICP-OES.

15. In vivo Anticancer Effect in 4T1 Tumor-xenografted Balb/c Mice

The 4-week-old female Balb/c Nude mice (~ 15 g) were obtained and raised at Laboratory Animal Center, Shanghai Medical College of Fudan University. To develop the animal tumor model, 4T1 xenografted tumors were generated in 4-week-old female Balb/c mice by subcutaneously (s.c.) injecting of 4T1 cells (1×10^6 cell/site, 100 μL saline) into the right rear legs of female Balb/c Nude mice. Once the tumor volume reached nearly 100 mm^3, the mice were randomly divided into three groups (n = 6). For the evaluation of the anticancer effect of DMSN-Au-Fe_3O_4 NPs, DMSN-Au-Fe_3O_4 NPs (100 μL saline solution, dose: 10 mg or 20 mg kg^{-1}) were intravenously injected into the mice in two groups respectively with 100 μL of saline solution injected into the other group as the control group. The length and width of tumors were measured by digital caliper every two days during half a month post injection. The tumor volume was calculated according to the following formula: tumor volume = $L \times W^2/2$, where L and W mean the length (mm) and width (mm) of tumor, respectively. The tumors were dissected in 24 h post-injection and sliced for further hematoxylin and eosin (H&E) staining assay for observing the structure and status of cells, terminal deoxynucleotidyl transferase mediated dUTP nick-end labeling (TUNEL) staining assay for detecting DNA fragmentation and Ki-67 antibody staining assay for detecting the growth fraction of cells.

16. Statistical Analysis

Quantitative data are expressed as mean ± s.d. Statistical comparisons were conducted by using Student's two-sided t-test as *$P < 0.05$ (significant), **$P < 0.01$ (moderately significant) and ***$P < 0.001$ (highly significant).

B: Supplementary figures and discussions

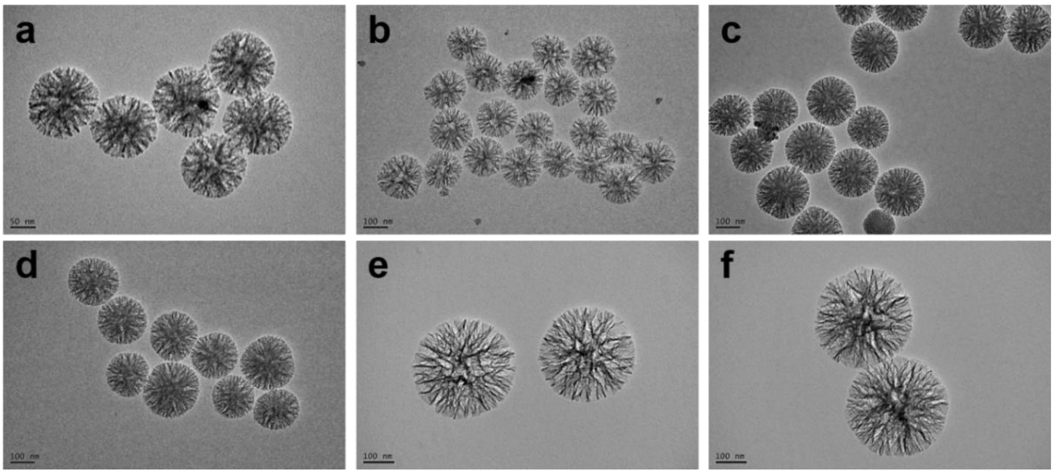

Figure S1. TEM images of DMSN NPs synthesized with varied CTAB/NaSal molar ratio and corresponding different size of (a) 1/0.25, 130 nm, (b) 1/0.5, 150 nm (c) 1/0.75, 180 nm (d) 1/1, 200 nm (e) 1/ 1.25, 280 nm and (d)1/1.5, 320 nm.

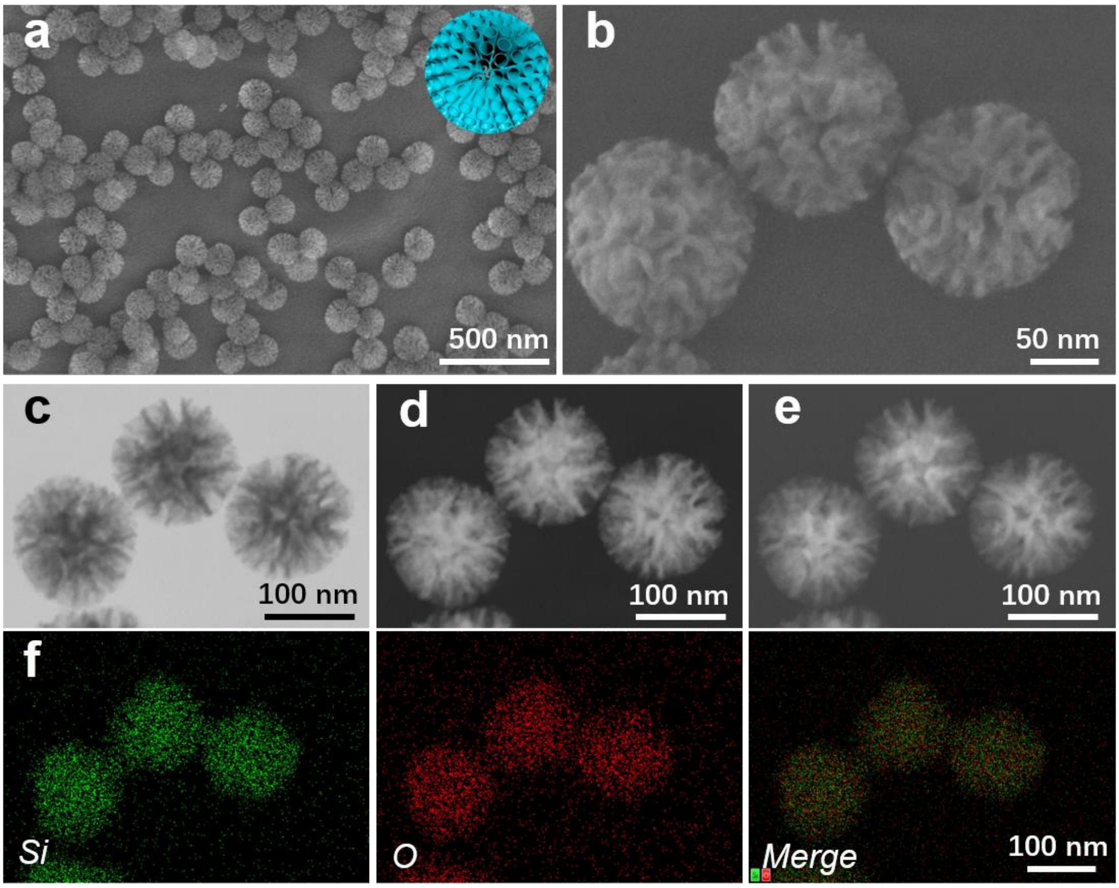

Figure S2. (a, b) SEM image, (c) bright-field TEM image, (d) dark-field TEM image, (e) HADDF image and (f) corresponding element mappings (for Si, O and merge) of DMSN NPs.

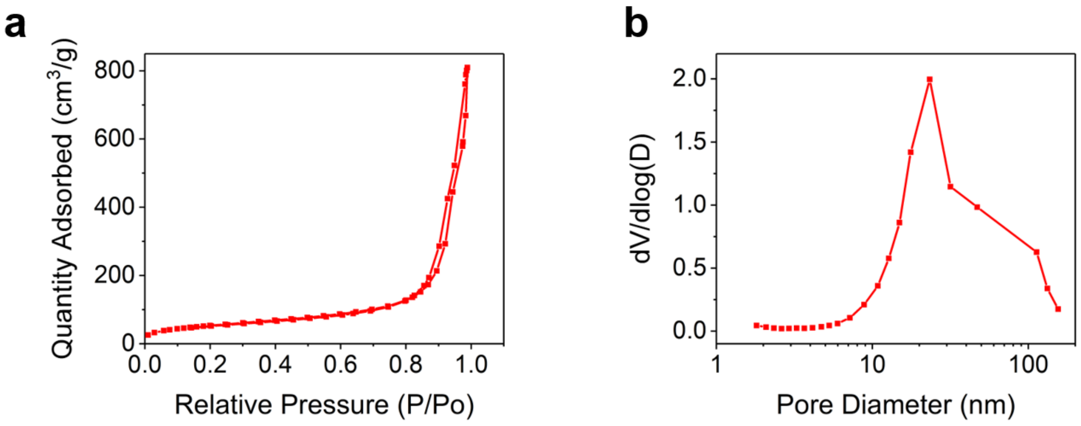

Figure S3. (a) N_2 absorption-desorption isotherm and (b) corresponding pore-size distribution of DMSN NPs.

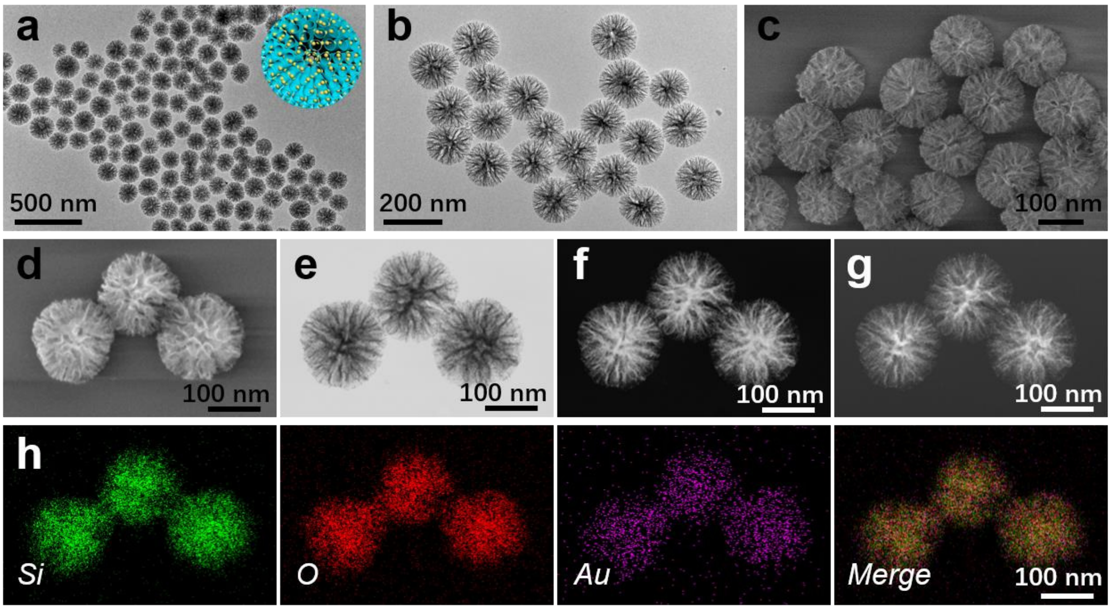

Figure S4. (a, b) TEM images, (c, d) SEM images, (e) bright-field TEM image, (f) dark-field TEM image, (g) HADDF image and (h) corresponding element mappings (for Si, O, Au and merge) of DMSN-Au NPs.

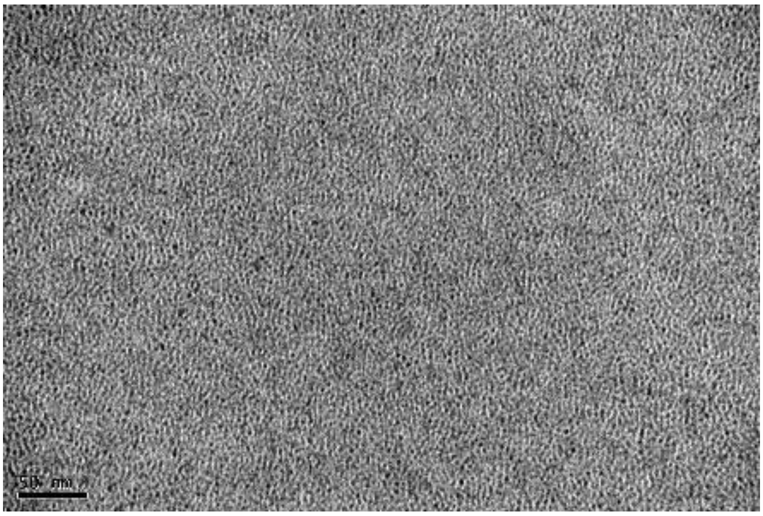

Figure S5. TEM image of monodispersed ultrasmall Fe_3O_4 NPs.

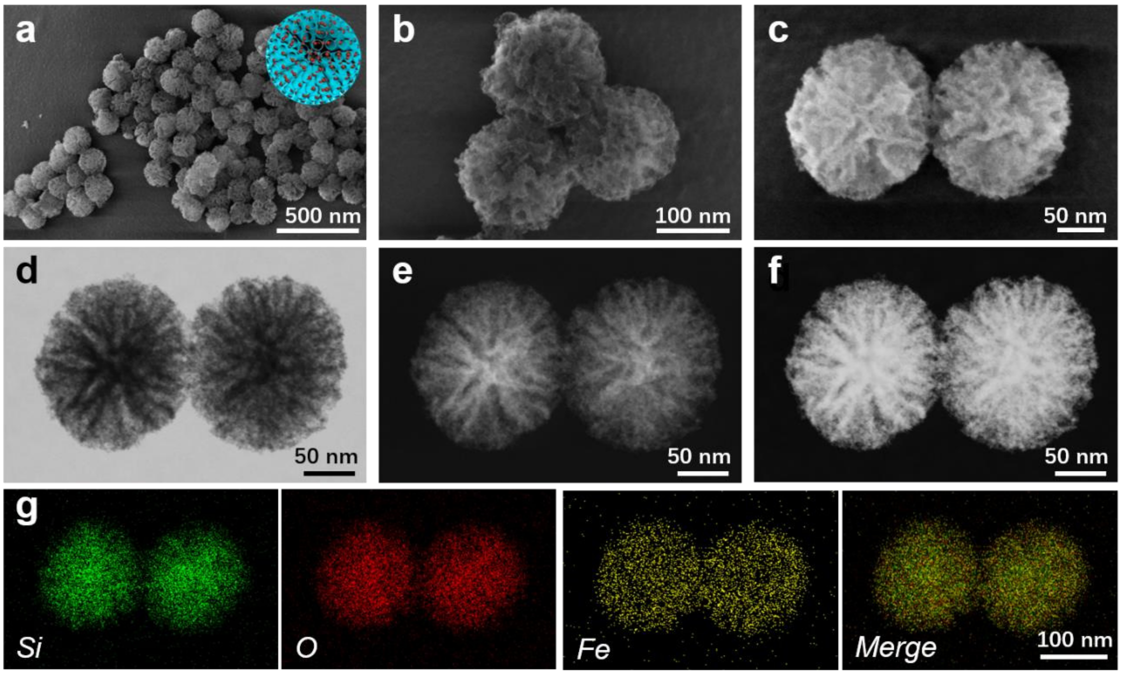

Figure S6. (a-c) SEM images, (d) bright-field TEM image, (e) dark-field TEM image, (f) HADDF image and (g) corresponding element mappings (for Si, O, Fe and merge) of DMSN-Fe_3O_4 NPs.

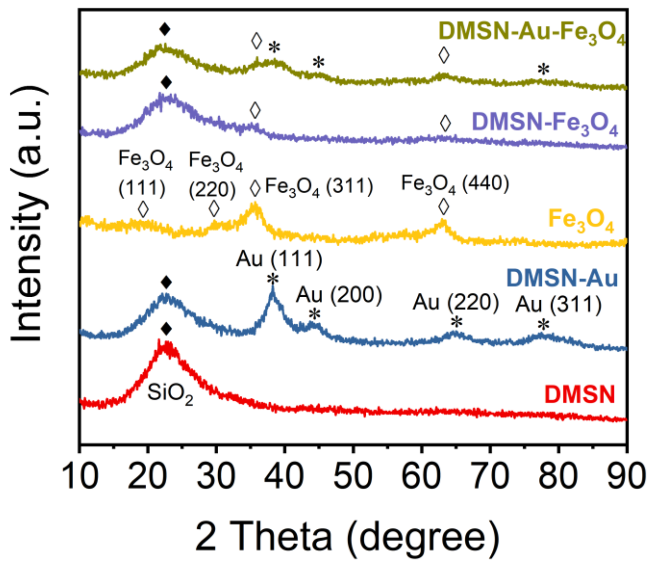

Figure S7. XRD patterns of DMSN, DMSN-Au, Fe_3O_4, DMSN-Fe_3O_4 and DMSN-Au-Fe_3O_4 NPs.

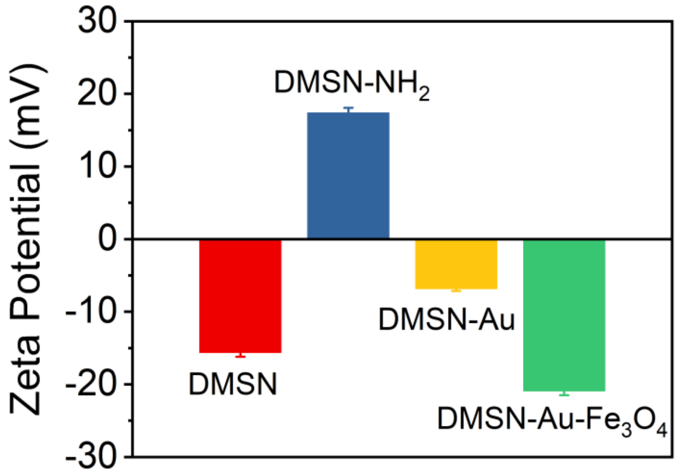

Figure S8. Zeta potentials of DMSN, DMSN-NH_2, DMSN-Au and DMSN-Au-Fe_3O_4 NPs.

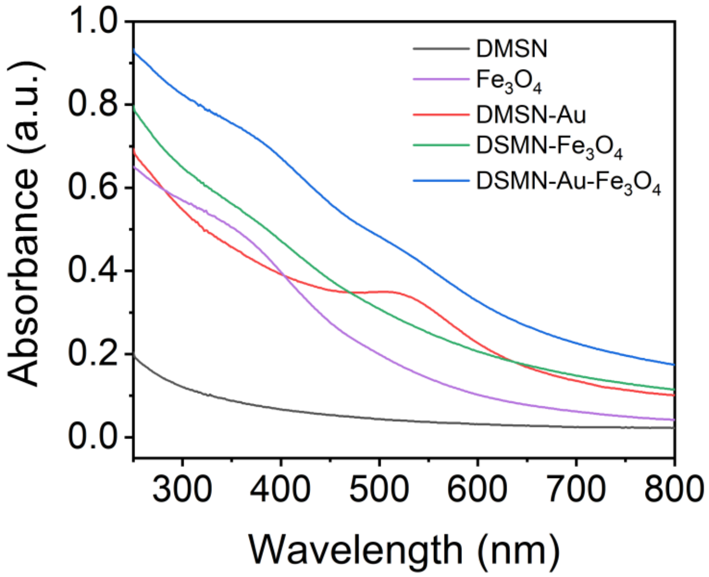

Figure S9. UV-vis spectra of DMSN NPs, Fe_3O_4 NPs, DMSN-Au NPs, DMSN-Fe_3O_4 NPs and DMSN-Au-Fe_3O_4 NPs.

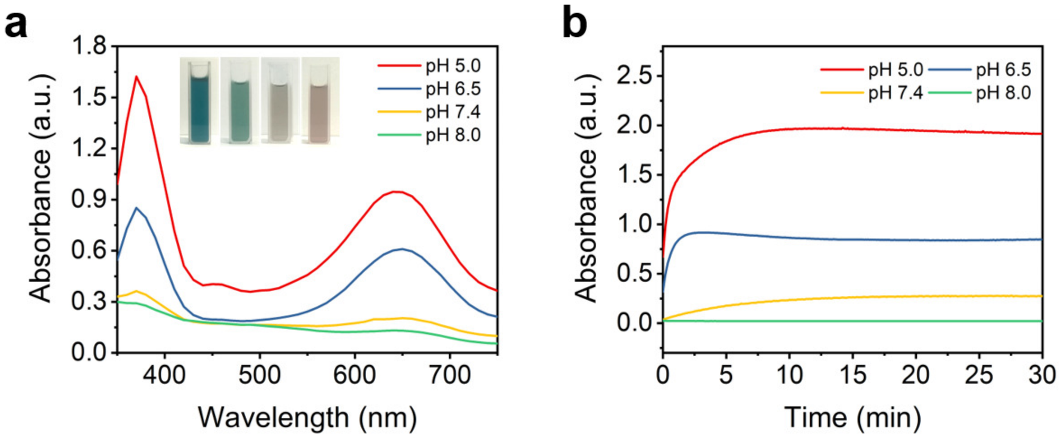

Figure S10. (a) UV-vis spectra of TMB as catalyzed by DMSN-Au-Fe_3O_4 NPs in the presence of glucose in reaction buffer at pH 5.0, 6.5, 7.4, and 8.0. Insets show the corresponding digital photos of each group. (b) Time-dependent absorbance changes at 652 nm as a result of the catalyzed oxidation of TMB by DMSN-Au-Fe_3O_4 NPs in the presence of glucose at pH 5.0, 6.5, 7.4, and 8.0.

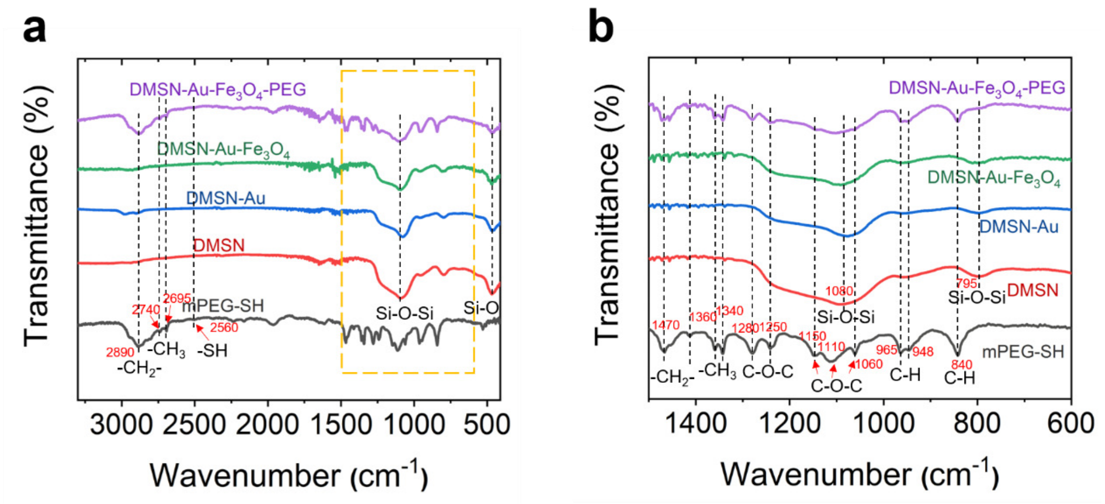

Figure S11. (a) FTIR spectra of mPEG-SH, DMSN NPs, DMSN-Au NPs, DMSN-Au-Fe_3O_4 NPs and DMSN-Au-Fe_3O_4-PEG NPs. (b) The enlarged region of the framed zone in panel a.

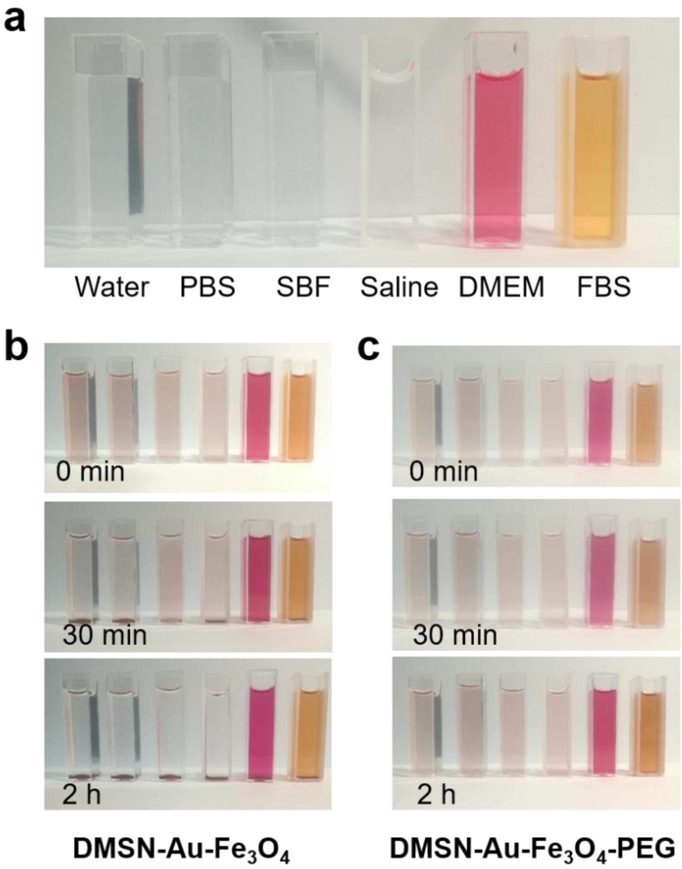

Figure S12. (a) Digital photographs of water, PBS, SBF, saline, DMEM and FBS. Digital photographs of the (b) DMSN-Au-Fe_3O_4 NPs and (c) DMSN-Au-Fe_3O_4-PEG NPs after dispersed in the above solutions for varied durations (0 min, 30 min and 2 h).

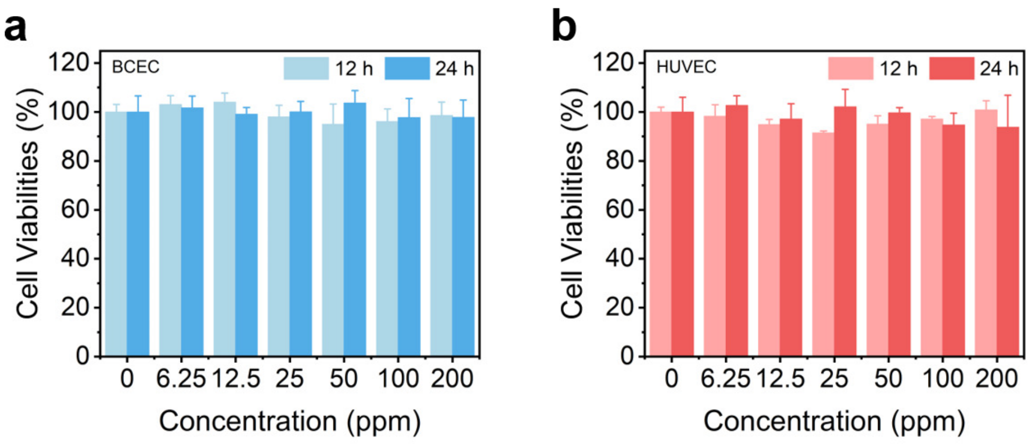

Figure S13. Relative viabilities of (a) BCEC and (b) HUVEC cells after being incubated with DMSN-Au-Fe_3O_4 NPs at varied concentrations (0, 6.25, 12.5, 25, 50, 100, and 200 μg mL^{-1}) for 12 h and 24 h.

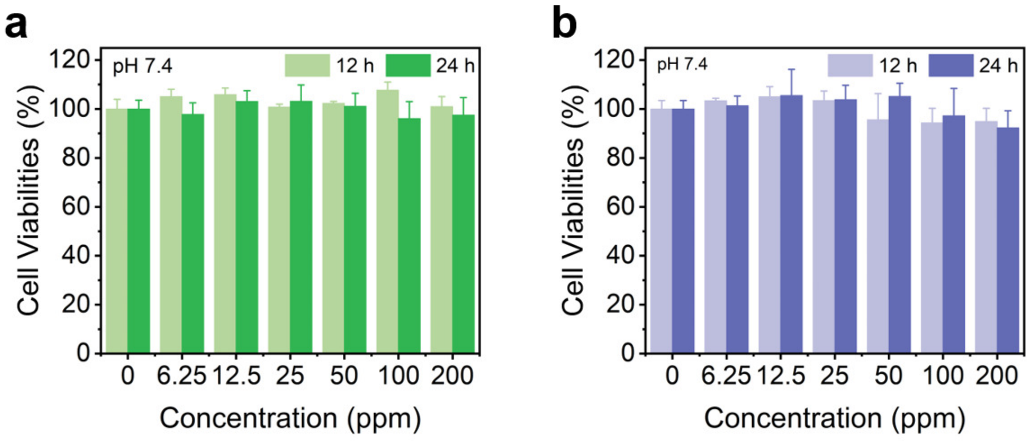

Figure S14. Relative viabilities of 4T1 cells after being incubated with (a) DMSN-Au NPs and (b) DMSN-Fe_3O_4 NPs at varied concentrations (0, 6.25, 12.5, 25, 50, 100, and 200 μg mL^{-1}) for 12 h and 24 h at pH 7.4.

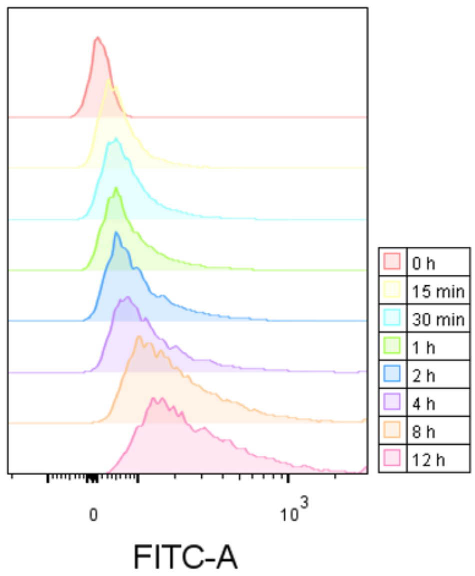

Figure S15. Flow cytometry analysis of 4T1 cells intracellular uptake of FITC-labeled DMSN-Au-Fe_3O_4 NPs after incubation for different durations (0, 15 min, 30 min, 1 h, 2 h, 4 h, 8 h and 12 h).

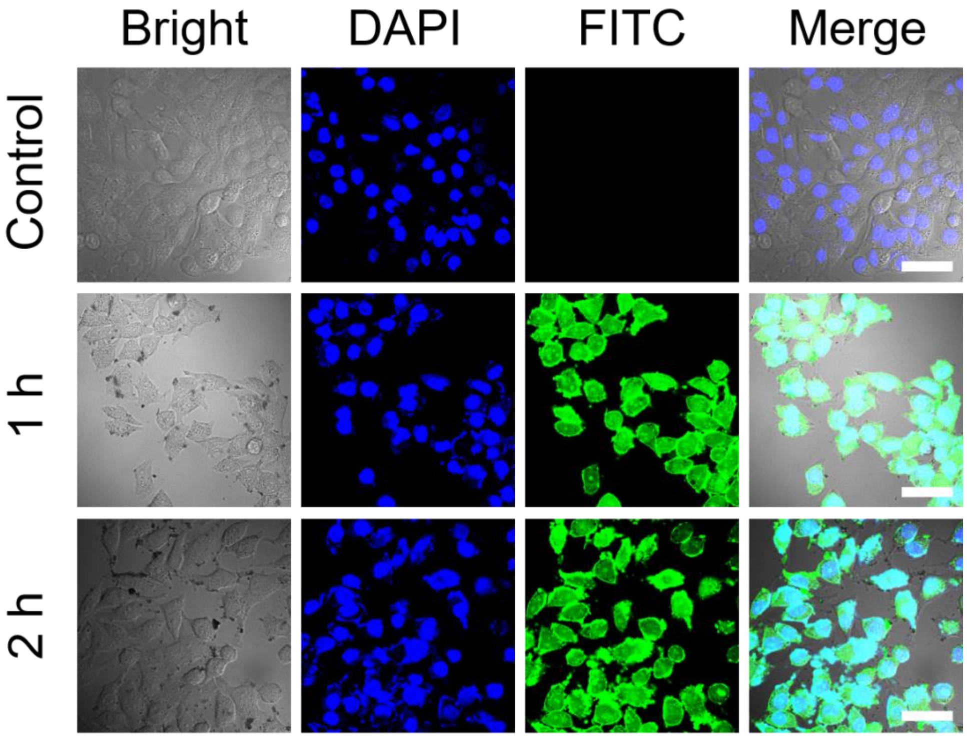

Figure S16. CLSM images of DAPI stained 4T1 cells incubated with FITC-labeled DMSN-Au-Fe_3O_4 NPs at the concentration of 50 μg mL^{-1} for 0, 1 and 2 h. Scale bar: 50 μm.

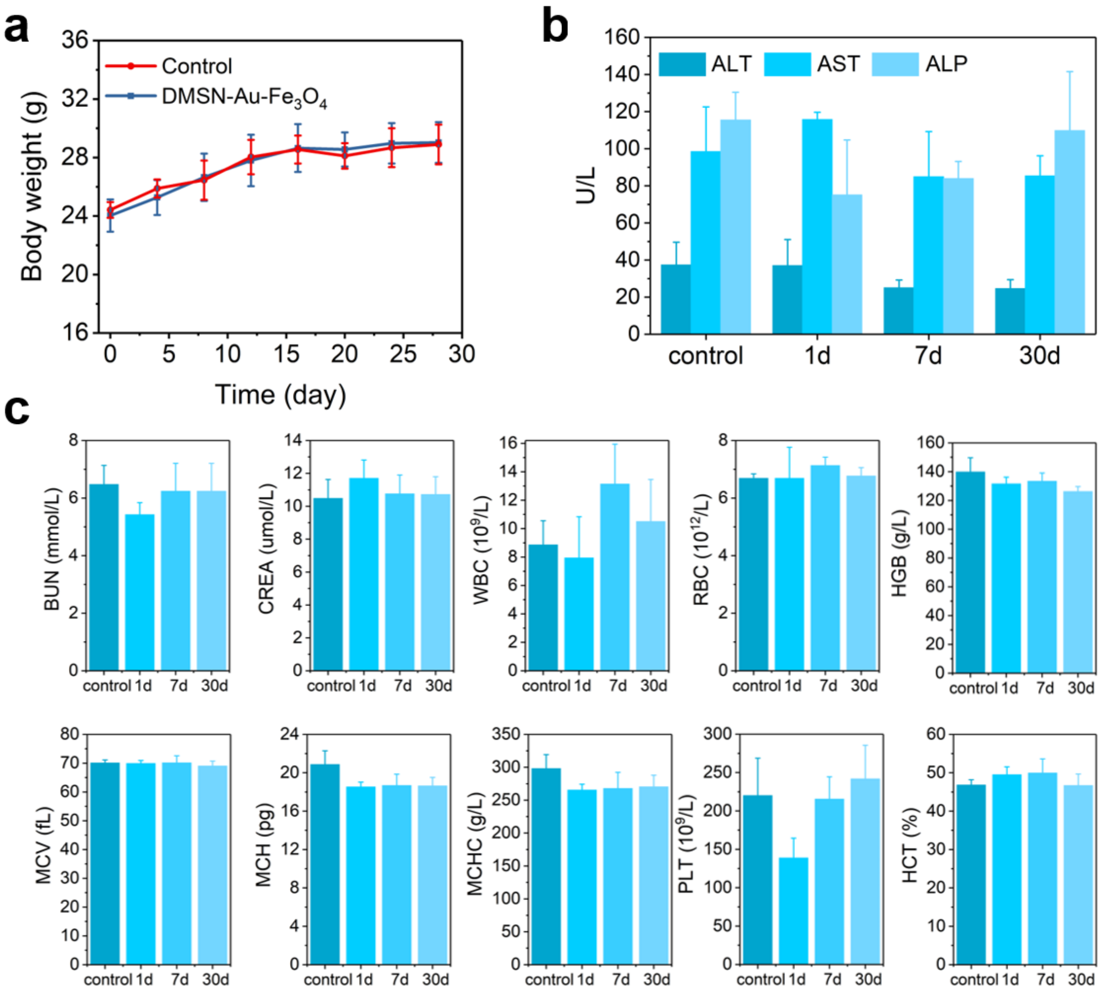

Figure S17. (a) Time-dependent body weight curves of healthy Kunming mice in 28 days after treatment with DMSN-Au-Fe_3O_4 NPs. (b) Blood-biochemical analysis of the body-weight of DMSN-Au-Fe_3O_4 NPs treated mice in 1, 7 and 28 days post-injection. (c) Hematological data of the mice intravenously injected with DMSN-Au-Fe_3O_4 NPs in 1, 7 and 28 days post injection.

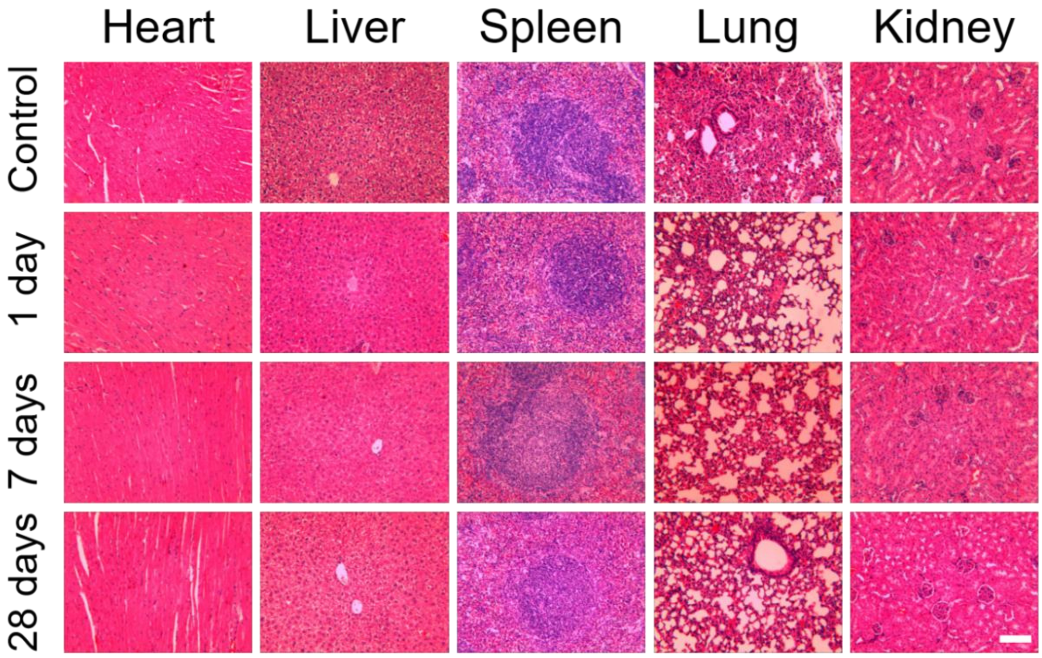

Figure S18. Histological assessments for the major organs (heart, liver, spleen, lung and kidney) of Kunming mice after treatment with DMSN-Au-Fe_3O_4 NPs in 1, 7 and 28 days post-injection. Scale bar: 100 μm.

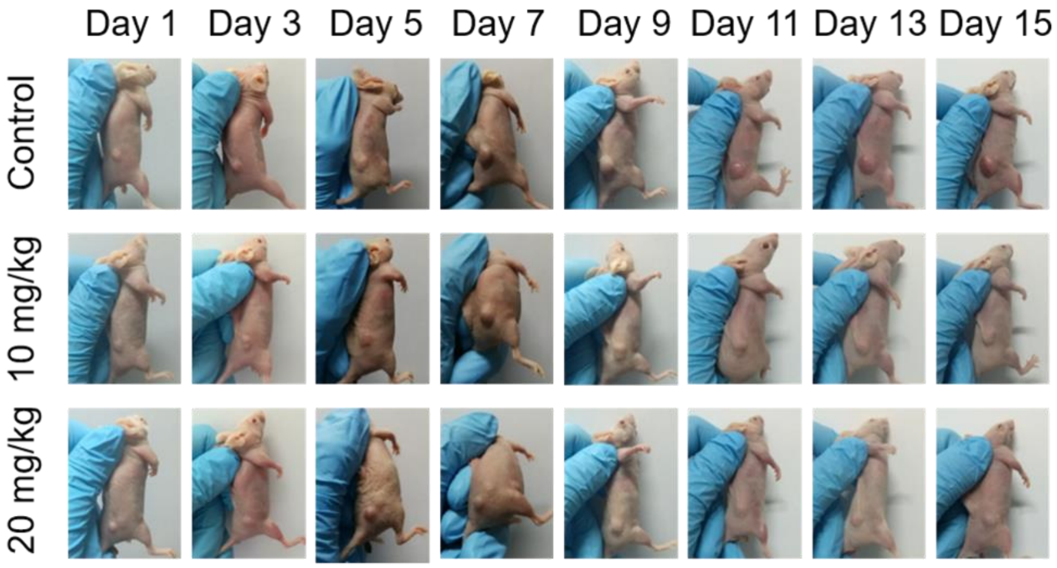

Figure S19. Digital photos of 4T1 tumor-bearing mice in different groups in 16 days after different treatments.

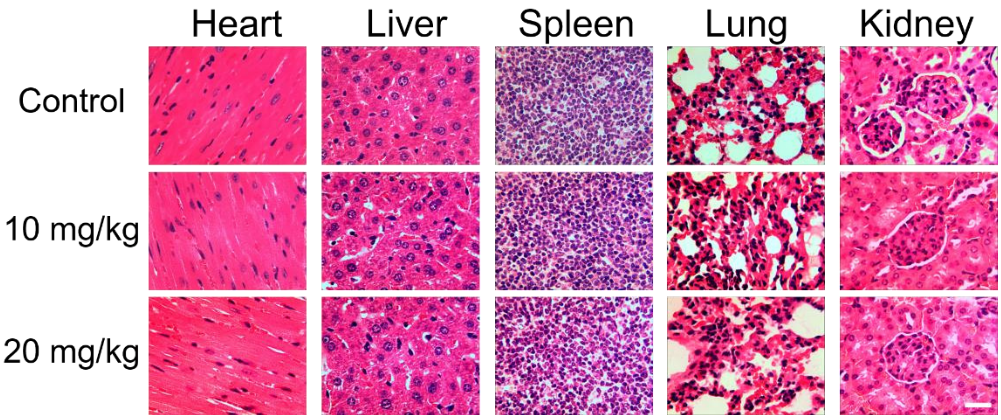

Figure S20. Histological assessments for the major organs (heart, liver, spleen, lung and kidney) of 4T1 tumor-bearing mice in two therapeutic groups and the control group after the therapeutic period. Scale bar: 25 μm.

Discussion S1. Systematic in vivo toxicity assessments.

The in vivo toxicity assessments of DMSN-Au-Fe_3O_4 composite nanocatalysts were conducted to guarantee their further potential clinical translation. Twenty SBF-level healthy female Kunming mice were randomly divided into four groups. After intravenous (i.v.) administration of DMSN-Au-Fe_3O_4 NPs (100 μL, dose: 20 mg kg^{-1}), three groups of Balb/c mice were sacrificed on the 1st, 7th, 28th day post injection, and the other group of untreated mice was set as the control group. No significant body-weight losses of mice in four groups were observed in the feeding duration of four weeks (**Figure S17a**). The standard blood biochemical indexes and normal hematology parameters were then examined, including alanine transaminase (ALT), aspartate transaminase (AST), alkaline phosphatase (ALP), blood urea nitrogen (BUN), creatinine (CREA), white blood cells (WBC), red blood cells (RBC), hemoglobin (HGB), mean corpuscular volume (MCV), mean corpuscular hemoglobin (MCH), mean corpuscular hemoglobin concentration (MCHC), platelets (PLT) and hematocrit (HCT). All these indexs in mice treated with DMSN-Au-Fe_3O_4 NPs in the groups of 1 day, 7 days and 28 days show no significant changes as compared to those in the control group (**Figure S17b, c**). These results imply that DMSN-Au-Fe_3O_4 NPs at such a high dose of treatment will not induce significant negative impact on the major blood indexes. The major organs of treated mice were harvested and sliced for histology analysis by hematoxylin and eosin (H&E) staining on the 1st, 7th, and 28th day post injection. (**Figure S18**). The stained tissue sections of heart, liver, spleen, lung and kidney in groups treated with DMSN-Au-Fe_3O_4 NPs for 1 day, 7 days and 28 days reveal no histological abnormalities in comparison to those of the control group, suggesting no significant acute and chronic pathological toxicity during the treatment periods. These results indicate the relatively high biosafety and biocompatibility of DMSN-Au-Fe_3O_4 NPs for further in vivo therapeutic application on combating cancer.

References:

[1] Y. Yang, S. Bernardi, H. Song, J. Zhang, M. Yu, J. C. Reid, E. Strounina, D. J. Searles, C. Yu, Chem. Mater. **2016**, 28, 704-707.

[2] J. Park, K. An, Y. Hwang, J.-G. Park, H.-J. Noh, J.-Y. Kim, J.-H. Park, N.-M. Hwang, T. Hyeon, Nat. Mater. **2004**, 3, 891-895.
